# Co-option of an ancestral peptidase controls developmental patterning in multicellular cyanobacteria

**DOI:** 10.1016/j.isci.2025.114265

**Published:** 2025-11-28

**Authors:** Xiaomei Xu, Anaïs Scholivet, Stéphanie Champ, Matthieu Bergé, Zulihumaer Yeerkenjiang, Jonas Desjardins, Yann Denis, Badreddine Douzi, Deborah Byrne, Emmanuel Talla, Amel Latifi

**Affiliations:** 1 Aix Marseille University, CNRS, LCB, Laboratoire de Chimie Bactérienne, Marseille, France; 2 Plateforme Transcriptomique, Aix-Marseille University, CNRS, IMM, Marseille, France; 3 Université de Lorraine, INRAE, DynAMic, 54000 Nancy, France; 4 Aix Marseille University, CNRS, Protein Expression Facility, Institut de Microbiologie de la Méditerranée Proteomic Platform, Marseille, France

**Keywords:** Developmental biology

## Abstract

Spatial patterning in multicellular organisms is commonly explained by Turing-type reaction-diffusion systems, but the maturation of diffusible inhibitors remains poorly understood. In the cyanobacterium *Nostoc* PCC 7120, nitrogen deprivation triggers a pattern of nitrogen-fixing heterocysts regulated by HetR and inhibitory peptides, including PatX. We uncover the post-translational mechanism controlling PatX maturation, demonstrating its export and subsequent processing by the peptidase PatP. We identify HRGTGR, a PatX-derived hexapeptide, as the direct inhibitor of HetR, linking maturation to suppressed differentiation. Genomic analyses reveal that *patP* is ancient and conserved across all cyanobacteria, predating the *patX-hetR* module found only in filamentous clades. We therefore propose that this ancient peptidase was co-opted to process a new ligand, transforming a proteolytic event into a spatial patterning mechanism. This repurposing parallels eukaryotic signaling, underscoring a universal principle in the emergence of multicellular organization and providing a model for how complex patterns evolve from “simple” components.

## Highlights

• PatX is exported to the periplasm and cleaved by PatP
• PatX-derived HRGTGR peptide binds and inactivates HetR, blocking heterocyst differentiation
• Ancestral cyanobacterial peptidase PatP predates both PatX and HetR
• The PatP-PatX-HetR module evolved in filamentous cyanobacteria

## Graphical abstract

**Figure:**
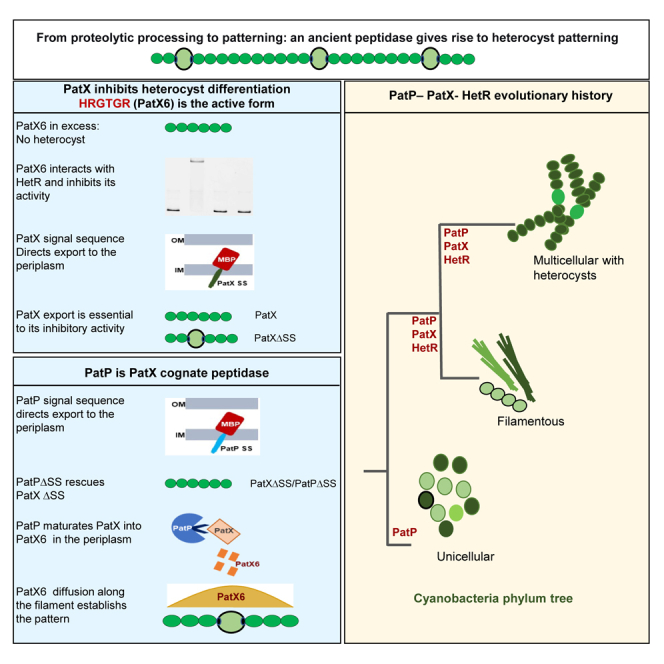

## Introduction

Multicellular organization and cellular diversity are complex mechanisms that allow organisms to develop specialized functions and adapt to environmental changes. Alan Turing’s reaction-diffusion model proposed that biological pattern formation in morphogenesis is driven by interacting components with different diffusion rates.^1^ Gierer and Meinhardt refined this theory, introducing an activator-inhibitor system with local self-enhancement and long-range inhibition to better explain biological development.^2^ Numerous studies have since confirmed the relevance of this model across diverse processes, including *Hydra* regeneration, leaf spacing, insect bristle and bird feather patterning, zebrafish pigment stripes, *Drosophila* segmentation, and cyanobacterial differentiation.^3^^,^^4^

Cyanobacteria constitute a monophyletic phylum of morphologically diverse bacteria capable of oxygenic photosynthesis.^5^ Their metabolism positions them as critical players in the biosphere due to their profound impact on global carbon and nitrogen cycles; several strains can fix atmospheric nitrogen.^6^ However, because the enzyme responsible for nitrogen fixation is inhibited by oxygen, diazotrophic cyanobacteria have evolved specialized strategies to reconcile oxygen-producing photosynthesis with nitrogen fixation, including spatial separation of the two antagonistic activities in multicellular strains.^7^

In many diazotrophic multicellular cyanobacteria, including the model organism *Nostoc/Anabaena* PCC 7120 (hereafter *Nostoc*), filaments consist solely of photosynthetic cells under nitrogen-replete conditions. During nitrogen deprivation, ∼10% differentiate into heterocysts—non-dividing cells that fix atmospheric nitrogen in a low-oxygen microenvironment.^7^^,^^8^^,^^9^ Oxygenic photosynthesis ceases within heterocysts, resulting in a micro-oxic environment suitable for the nitrogenase complex, which is specifically expressed in these cells. New heterocysts form midway between existing ones as vegetative cells grow, sustaining a dynamic pattern of ∼10–12 vegetative cells between heterocysts.^9^

Heterocyst differentiation involves four stages—induction, pattern formation, commitment, and morphogenesis—regulated by signaling cascades involving the master transcriptional regulator HetR.^10^ Nitrogen starvation triggers 2-oxoglutarate (2-OG) accumulation, activating NtcA to upregulate *hetR*.^11^^,^^12^ Deletion of *hetR* prevents differentiation, while its overexpression induces contiguous heterocysts formation even under non-permissive conditions.^10^ HetR initiates a negative feedback loop by activating *patS* expression.^13^ The resulting 17-amino-acid product is processed into the signaling peptide PatS5 (RGSGR) or PatS6 (ERGSGR), which binds to HetR to inhibit its DNA-binding function.^13^^,^^14^^,^^15^

Exogenous PatS5 or PatS6 peptides suppress differentiation, indicating that they act as a diffusible signal.^13^^,^^14^ PatS6 acts as an inhibitory morphogen, diffusing along the filament to suppress HetR activity in adjacent vegetative cells, preventing their differentiation.^16^^,^^17^^,^^18^ This HetR/PatS interaction exemplifies the local activation/long-range inhibition model of pattern formation.^19^^,^^20^ The RGSGR pentapeptide, critical for PatS function, is conserved in HetN—another inhibitor that maintains heterocyst patterning—which suggests that, by analogy to PatS, the peptide resulting from HetN processing also acts through diffusion along the filament.^17^^,^^21^^,^^22^ Differentiating cells achieve immunity to self-inhibition through HetL, which binds HetR and blocks inhibitory morphogen binding.^2^^,^^4^

While *hetR* is conserved across heterocyst-forming cyanobacteria, *patS* and *hetN* are not,^23^ implying the existence of additional inhibitory factors. Recently, PatX, containing the HRGTGR hexapeptide, was identified as coextensive with HetR in filamentous cyanobacteria.^23^ Phylogenetic studies indicate that the PatX/HetR pair was present in multicellular cyanobacteria before PatS, HetN, and the emergence of heterocyst development,^23^ suggesting a role in early cyanobacterial multicellularity. *patX* expression is induced 6–8 h post-nitrogen starvation in prospective heterocysts under NtcA and HetR control.^23^ PatX overexpression, like that of PatS and HetN, inhibits heterocyst differentiation.^23^^,^^24^

In the presence of combined nitrogen, a *patX* single mutant—whose *patS* and *hetN* genes are wild-type—forms heterocysts, revealing that PatX acts as a negative regulator of cell differentiation.^25^ Under nitrogen-fixing conditions, however, this single mutant displays a normal heterocyst pattern, whereas simultaneous inactivation of *patS* and *hetN* in a *patX*-deficient background triggers rapid, synchronous differentiation.^25^ This suggests that the inhibitory effect of PatX is, at the time of observation, masked by the presence of the other two inhibitors, PatS and HetN. Given that PatX is present in filamentous strains not forming heterocysts,^23^ it has been proposed that its original function may have been to control patterned behaviors associated with multicellularity, such as necridia (ghost cells) formation.^23^ This role could later have been co-opted to suppress supernumerary heterocysts in lineages that evolved nitrogen-fixing differentiation.

PatX N-terminus contains a Sec-machinery signal sequence, suggesting Sec-dependent translocation outside the cytoplasm.^26^ The molecular mechanism of PatX, the functional role of its signal sequence, and the process by which PatX is cleaved into its active form remain key unanswered questions.

Here, we demonstrate that PatX is translocated outside the cytosol and is cleaved by the peptidase Alr1666 (PatP). We also show that the 6-amino-acid PatX-derived peptide (HRGTGR) binds HetR and inhibits its activity. Phylogenomics indicate that PatP emerged early in cyanobacterial evolution, before PatX and HetR—their later co-option of this ancient protease converted a proteolytic event into the lineage-specific Turing mechanism patterning heterocysts.

## Results

### PatX directly inhibits HetR activity to modulate heterocyst formation in *Nostoc*

**Figure 1:**
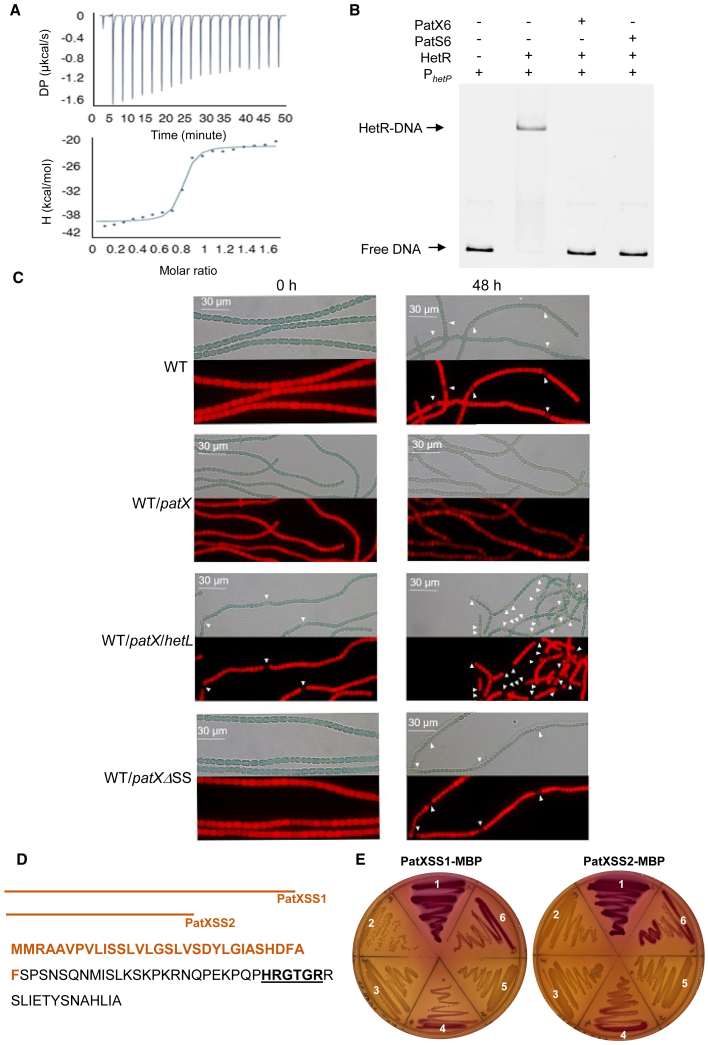
PatX inhibits HetR and contains a functional periplasmic export signal (A) ITC experiment to analyze HetR interaction with PatX6. The respective upper panel shows heat exchange upon ligand titration, and the bottom panel shows integrated data with binding isotherms (solid line) fitted to a single-site binding model. The constant heat dilution was removed before the integrated binding isotherms. The titrant PatX6 (200 μM) was titrated into a cell-containing 22 μM HetR at 25°C. (B and C) (B) EMSA assay of HetR (1 μM) with the *hetP* promoter (50 nM) in the presence of PatX6 or PatS6 (1 μM). The *hetP* promoter incubated alone served as a negative control (free DNA). HetR-DNA indicates the complex formed by HetR and the promoter (C). Bright-field (top) and autofluorescence of photosynthetic pigments (bottom) microscopic images of the indicated strains. The scale bar is labeled in micrometers (μm). *patX* and *hetL* overexpression was induced with 3 μM copper. Heterocysts are indicated by white arrows. Time denotes hours post combined nitrogen starvation. Scale bars, 30 μm. (D) PatX sequence with the two predicted Sec-type signal sequences shown in brown. The inhibitory hexapeptide of PatX is indicated in bold and underlined. (E) Phenotypes on MacConkey plates of *E. coli* MG1655 derivatives. Sector 1: wild-type. Sector 2: *malE* mutant. Sectors 3–6: *malE* mutant complemented with plasmids: sector 3, empty vector (p33tac); sector 4, p33tac-*malE* (full length); sector 5, p33tac-*malE*ΔSS; sector 6, p33tac-*malE* fusions with PatX signal sequences (SS1 on left, SS2 on right). The two PatXSS used to construct the MBP fusions are indicated in (D). Red color indicates MBP periplasmic export.

The finding that ectopic expression of *patX* or exogenous application of the PatX6 peptide suppresses cellular differentiation in *Nostoc*^20^^,^^21^ implies that PatX may act analogously to PatS by inhibiting HetR activity. To investigate this possibility, we assessed whether a cytosolic PatX protein (without the putative signal sequence) and/or the PatX6 hexapeptide (HRGTGR) directly interacts with HetR. Bacterial two-hybrid assays (T18/T25 CyaA reconstitution^27^) revealed no interaction between HetR and signal-sequence-less PatX (β-gal activity indistinguishable from parental vectors lacking inserts; Figure S1A). An isothermal titration calorimetry (ITC) assay was conducted to analyze the potential interaction between HetR and PatX6. Results showed that PatX6 binds tightly to HetR, with an estimated dissociation constant (*K*_D_) for the HetR-PatX6 interaction in the nanomolar range (88 nM) (Figure 1A).

To determine whether PatX6 inhibits HetR’s DNA-binding activity, we performed electrophoretic mobility shift assays (EMSAs) using the *hetP* promoter, a known HetR target gene.^11^^,^^12^ While HetR bound the promoter alone, addition of either PatS6 (positive control) or PatX6 abolished this interaction (Figure 1B), demonstrating direct inhibition of HetR-DNA binding. To validate PatX’s inhibition of HetR *in vivo*, we exploited HetL, a known immunity protein that protects HetR from peptide inhibitors inside the heterocyst.^24^ Overexpression of *patX* blocked heterocyst formation, but co-expression with *hetL* restored differentiation—even under repressive conditions (BG11, *T* = 0 h; Figure 1C). This genetic suppression confirms that PatX targets HetR activity *in vivo*.

### The extracytosolic translocation of PatX is essential for its activity

SignalP 5.0 predicted two Sec-type signal peptides in PatX (SS1: residues 1–20; SS2: residues 1–31; Figure 1D). To test its possible translocation to the periplasm, we fused each peptide to maltose-binding protein lacking its signal sequence (MBPΔSS) and transformed the constructs into a *malE*-deficient *Escherichia coli* strain. Functional export was scored on MacConkey-maltose plates: red colonies indicate periplasmic MBP activity and maltose fermentation, and white colonies indicate retention in the cytosol.^28^ Both PatX-SS1-MBPΔSS and PatX-SS2-MBPΔSS yielded red colonies comparable to wild-type controls, whereas the insert-less vector or MBPΔSS alone remained white (Figure 1E; compare sectors 1 and 6). The shorter SS2 was therefore used as the definitive PatX signal sequence in subsequent experiments.

To test whether export outside the cytosol is required for PatX activity in *Nostoc*, we deleted its signal sequence (*patXΔSS*) and compared the inhibitory potency to full-length *patX* under the copper-inducible promoter *petE*. qRT-PCR confirmed 10- to 30-fold overexpression for both constructs (Figure S1B).

Notably, while heterocyst differentiation was completely inhibited in the WT/*patX* strain due to PatX activity, the WT/*patXΔSS* strain retained cell differentiation capability, producing heterocysts at a frequency of 7% (Figure 1C; Table S1). This result demonstrates that PatX must be translocated to the periplasm to inhibit heterocyst differentiation.

### Identifying potential peptidases for PatX maturation in *Nostoc*

Our data suggest that PatX is processed into its hexapeptide outside the cytosol. We therefore hypothesized that the responsible peptidase is also extracytosolic. To identify candidate proteases, we first selected those predicted to be exported and then analyzed their expression profiles.

In diderm bacteria (including *Nostoc*), Sec-type signal peptides direct proteins to the periplasm.^29^ Using the MEROPS database, we therefore screened the *Nostoc* genome for genes encoding peptidases bearing either motif.

The eight sorted proteins included six with Sec-type signal peptides and two with TAT-type sequences. Among these eight proteins, six have predicted functions involved in cell wall remodeling and cell division (Table S2). The remaining two proteins, Alr1666 and All2656, are classified as hypothetical proteins (Table S2) and were thus selected for further analysis.

**Figure 2:**
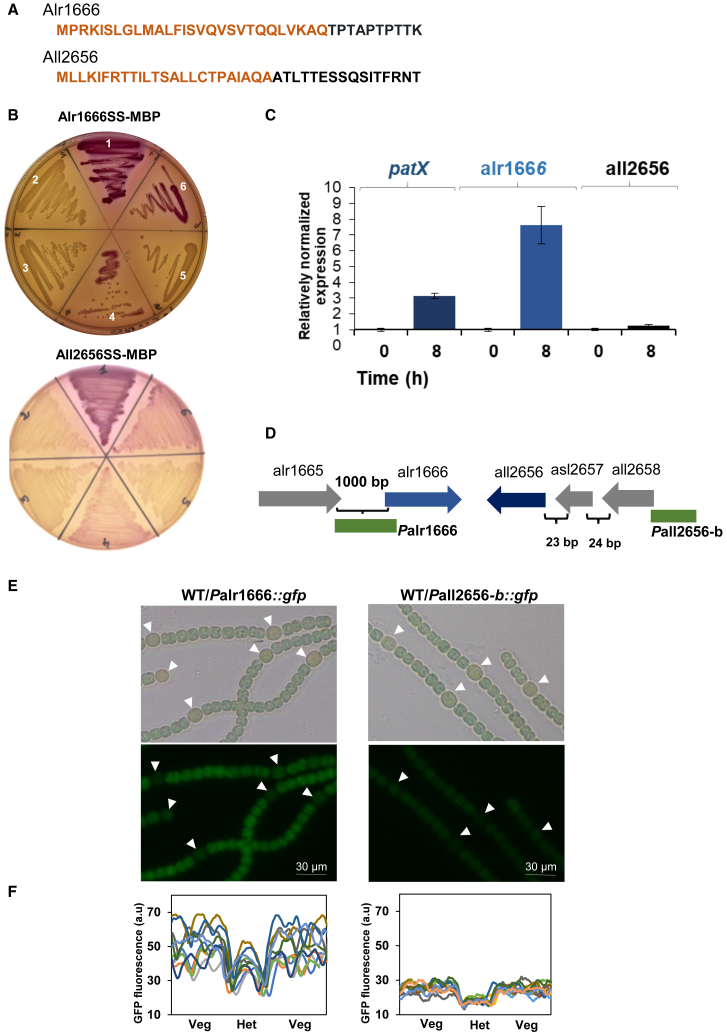
Transcription profile of alr1666 and all2656 and periplasmic translocation of their encoded proteins (A) The predicted Sec-type signal sequences of All2656 and Alr1666 are shown in brown. (B) MBP localization based on MacConkey assay. Sector 1: wild type *E. coli* MG1655; sector 2: *E. coli* MG1655 *malE* mutant; sector 3: the *malE* mutant with p33tac plasmid; sector 4: the *malE* mutant with p33tac-*malE* full length; sector 5: the *malE* mutant with p33tac-*malEΔSS*; sector 6: the *malE* mutant with p33tac-alr1666*SS*-*malE* (upper) or p33tac-all2656SS-*malE* (lower). (C) Gene transcription analyzed by qRT-PCR. Each sample was measured in triplicate, and data are represented as mean ± SEM indicated by error bars. The indicated time refers to the incubation period after combined nitrogen starvation. The values obtained at time 0 were set to 1. (D) Genomic organization of alr1666 and all2656 loci. Intergenic region lengths are shown in base pairs (bp). Gene segments fused to GFP are highlighted in green. (E) Microscopic bright field images (top), and GFP-fluorescence images (bottom) of the indicated *Nostoc* strains 24 h after combined nitrogen starvation. White arrows indicate heterocysts. Scale bars, 30 μm. (F) GFP fluorescence quantification. Fluorescence intensity profiles were measured along 10 filament segments (each containing one heterocyst [Het] and three vegetative cells [Veg] per side) using ImageJ. Each curve represents the intensity distribution for one filament. Intensity is in arbitrary units (a.u.).

To investigate the ability of the Alr1666 and All2656 signal sequences to drive periplasmic translocation in *E. coli*, we conducted MalE fusion assays. Proteins MalEΔSS, fused with the predicted signal sequences of Alr1666 or All2656 (Figure 2A), localized to the periplasm (Figure 2B), indicating that both sequences are functional signal peptides.

Quantitative RT-PCR revealed that alr1666 transcription rises ∼7-fold within 8 h of nitrogen removal, coincident with a 3-fold induction of *patX*, while all2656 levels remained unchanged (Figure 2C), pointing to alr1666 as a nitrogen-responsive gene.

To map cell-type expression, we fused each candidate peptidase promoter region to the GFP coding sequence. For alr1666, a 1 kb fragment upstream of the start codon (Palr1666) was used (Figure 2D); for all2656, two constructs were tested because the gene may lie within an operon with all2657–all2658 (Figure 2D). The first (Pall2656a, 1 kb upstream of all2656) gave no fluorescence (Figure S2B), so subsequent analysis used the second construct (Pall2656b, 1 kb upstream of all2658) that contains the predicted operon promoter (Figure 2D).

Both Palr1666 and Pall2656b fusions produced detectable GFP. Pall2656b drove faint, uniform fluorescence in BG11 (Figure S2B) and BG11_0_ (Figure 2E). In contrast, Palr1666 produced stronger signals in both BG11 (Figure S2B) and BG11_0_ (Figure 2E); but showed lower fluorescence in heterocysts (Figures 2E and 2F). Unlike the qRT-PCR approach, the GFP fusion assay does not allow for relative quantification between different growth conditions. This inherent limitation of the method likely explains why we observe no significant difference in signal between the BG11 and BG11_0_ conditions.

### Establishing Alr1666 (PatP) as a key peptidase in PatX maturation

To assess whether All2656 or Alr1666 mediates PatX maturation into its active peptide, we hypothesized that peptidase mutant(s) would phenocopy *patX* deletion. While *patX* inactivation alone does not alter heterocyst formation, a conditional *patS patX* mutant exhibits excessive differentiation, including contiguous heterocysts and necridia.^25^ We thus predicted that *patS*-peptidase double mutants would resemble the *patS patX double mutant*.

Using CRISPR-Cpf1, we deleted the genes all2656 and alr1666 in *Nostoc*, and—because cyanobacteria contain many identical genome copies—we confirmed by PCR/qRT-PCR that every copy had acquired the deletion (fully segregated Δall2656 and Δalr1666 mutants; Figures S3A–S3C) before we subsequently introduced the Δ*patS* mutation into these mutant backgrounds.

**Figure 3:**
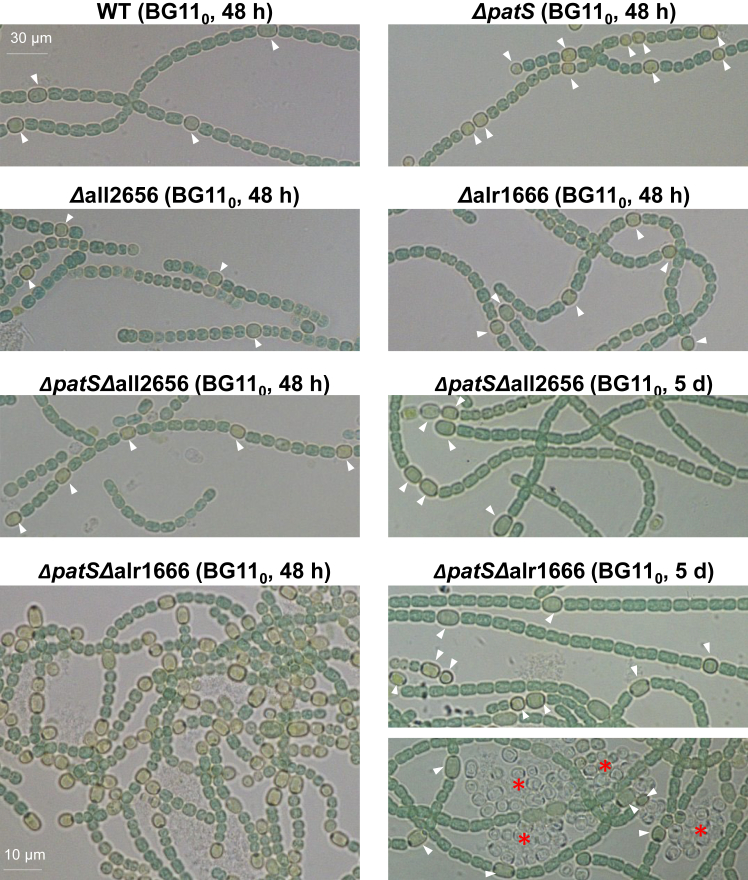
Heterocyst formation proficiency of *patS* mutants, peptidase mutants, and corresponding double mutants Bright-field microscopy images of the indicated *Nostoc* strains 48 h and 5 days after transfer from BG11 plates to BG11_0_ liquid medium. The scale bar for all panels is 30 μm, except for the panel marked with a 10 μm scale bar. White arrows indicate heterocysts. Asterisks indicate necridia.

As expected, the Δ*patS* mutant showed an irregular heterocyst pattern within 48 h of nitrogen deprivation; multiple contiguous heterocysts were observed (Figure 3), and the frequency of heterocysts was higher than that of the wild type strain (12% versus 9%, Table S1). Consistent with prior reports,^25^ the Δ*patX* mutant formed heterocysts in the presence of combined nitrogen (BG11 liquid medium) (Figure S3D). Single peptidase mutants showed a wild-type-like heterocyst pattern (Figure 3). In the Δ*patS*Δ*all2656* strain, heterocyst frequency (12%) mirrored Δ*patS* alone. Strikingly, the Δ*patS*Δ*alr1666* double mutant displayed excessive differentiation (37% heterocysts at 48 h; Figure 3; Table S1) and necridia by day 5 (Figure S3D), resembling the *patS patX* double mutant strain.

### Co-localization of PatP and PatX is essential for PatX processing and differentiation inhibition

To test whether Alr1666 matures PatX, we exploited the fact that full-length PatX over-expression blocks differentiation, whereas a cytosolic variant (PatXΔSS, lacking the Sec signal peptide) did not, indicating functional impairment (Figure 1C)—presumably because the cytoplasm lacks the necessary processing enzymes. We therefore co-expressed cytosolic Alr1666 (Alr1666ΔSS) with PatXΔSS; if Alr1666 is the relevant peptidase, co-expression should restore processing and release inhibitory peptides sufficient to block differentiation.

**Figure 4:**
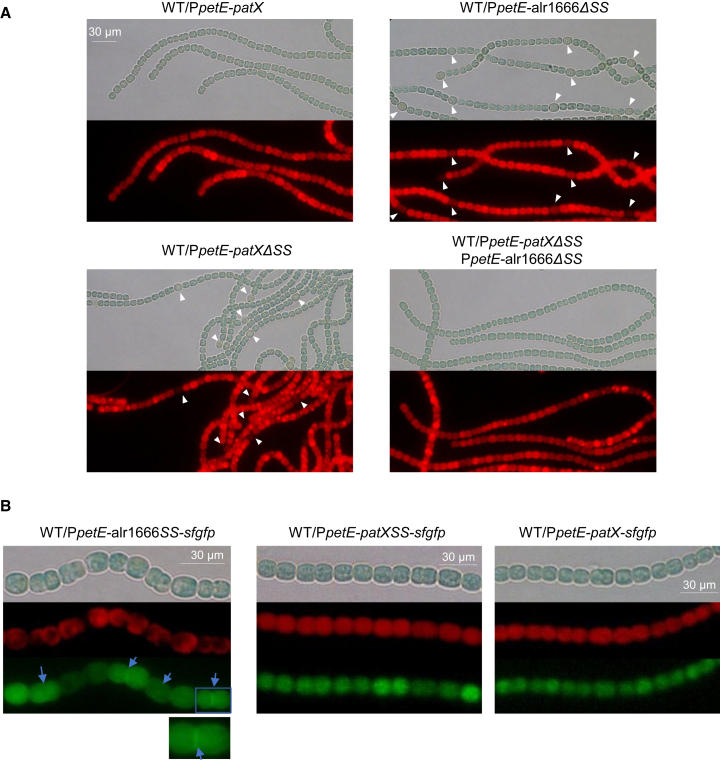
The co-localization of Alr1666 and PatX is required for PatX activity (A) Microscopic bright field images (top) and autofluorescence images (bottom) of the indicated *Nostoc* strains 24 h after nitrogen stepdown. *alr1666* and *patX* overexpression was induced with 3 μM copper. Heterocysts are indicated by white arrows. The absence of heterocysts indicates that PatX activity effectively blocks heterocyst development. (B) *Localization* of sfGFP fusion proteins in the indicated *Nostoc* strains. For each strain, microscopic images are shown: bright-field (top), autofluorescence of photosynthetic pigments (middle), and GFP fluorescence (bottom). The scale bars are indicated. Two hundred eighty fluorescent cells from three independent experiments of each strain were analyzed, and the representatives are shown. Left: PatPSS-sfGFP (imaged with 100x lens). The protein localizes to the cell periphery, particularly at the junction between cells (see magnified view), with some diffuse cytoplasmic fluorescence also present. A 2× digital magnification of the boxed region is shown below; blue arrows highlight the peripheral GFP signal. Middle: PatXSS-sfGFP (imaged with 60x lens). Only a diffuse cytosolic GFP signal was detected. Right: PatX-sfGFP (imaged with 60x lens). Only a diffuse cytosolic GFP signal was detected. Scale bars, 30 μm.

Both genes (*patXΔSS* and alr1666ΔSS) were expressed under the copper-inducible *petE* promoter, with qRT-PCR showing ≥8-fold overexpression (Figure S4). Strikingly, the double-overexpression strain completely abolished heterocyst formation (Figure 4A; Table S1), demonstrating that Alr1666 processes PatX in the cytoplasm to generate the inhibitory signal, thus identifying it as PatX’s cognate peptidase.

In cyanobacteria, exported proteins are delivered to one of two non-cytoplasmic compartments—the periplasm or the thylakoid lumen—whose Sec and Tat machineries recognize distinct signal-peptide signatures (discussed later). The *E. coli* Sec system accepts only periplasmic-targeting peptides,^30^ and both PatX and Alr1666 signal sequences drive Sec-dependent export to the *E. coli* periplasm (Figures 1E and 2B). We therefore expected the same destination in *Nostoc*. A recent spatial-proteomics survey that classified periplasmic versus thylakoid signal sequences independently listed Alr1666 as periplasmic, strengthening this prediction.^31^

To visualize export *in vivo*, we fused super-folder GFP (sfGFP) to the Alr1666 signal peptide and to full-length PatX or its signal peptide alone. All fusions were expressed from a copper-inducible *petE* promoter on a replicative plasmid. In live filaments, Alr1666-sfGFP produced a bright fluorescent rim between cells, indicative of periplasmic localization (Figure 4B); however, most of the fluorescence was seen inside the cytosol—likely due to protein overexpression. Occasional dim cells likely reflect expected plasmid heterogeneity.^32^ PatX-sfGFP remained cytosolic (Figure 4B), but this is not incompatible with periplasmic targeting: the sfGFP folds rapidly and can trap fusion partners in the cytoplasm when translocation is post-translational.^33^ Because both signal peptides nevertheless direct robust export in *E. coli*, the collective data support a periplasmic location for Alr1666 and PatX in *Nostoc*.

### *Ex vivo* cleavage assay confirms Alr1666 as PatX cognate peptidase

A dual-expression system was developed to evaluate Alr1666-mediated cleavage of PatX, overcoming obstacles related to the purification of the small (56 aa) PatX protein and the toxicity of Alr1666 overproduction. In this system, TrxA-PatXΔSS-MBP fusion, expressed from the IPTG-inducible *ptac* promoter, was co-expressed with Alr1666ΔSS-V5 under the control of the arabinose-inducible *pBAD promoter*. Cleavage and Alr1666 production were assessed via western blot using anti-TrxA and anti-V5 antibodies, respectively.

**Figure 5:**
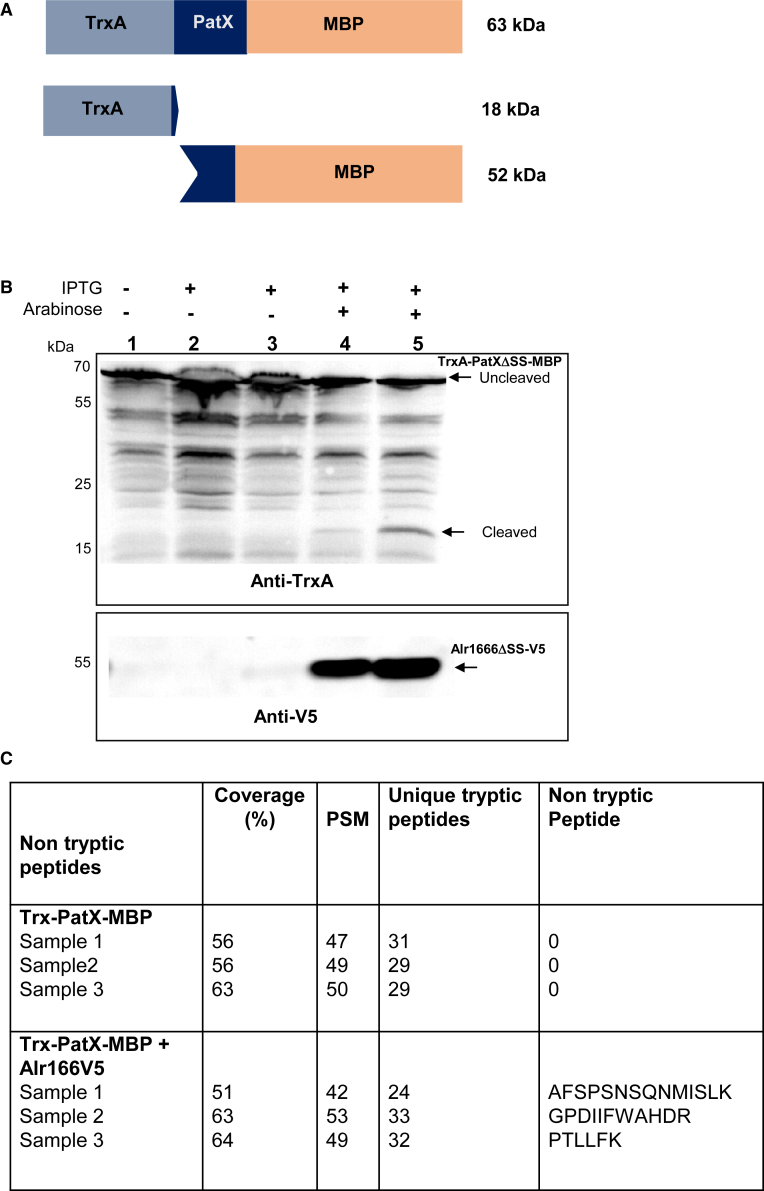
*Ex vivo* cleavage assay of TrxA-PatXΔSS-MBP by the putative peptidase Alr1666 (A) Schematic of the TrxA-PatXΔSS-MBP fusion protein construct, shown before and after potential cleavage by Alr1666. (B) Immunoblot analysis of cleavage assays. Expression of *trxA-patXΔSS-malE* was induced with IPTG; expression of alr1666-V5 was induced with arabinose. The blot was probed with anti-TrxA antibodies (top panel) to detect the full-length TrxA-PatXΔSS-MBP fusion and any TrxA-PatX cleavage product, and with anti-V5 antibodies (bottom panel) to confirm Alr1666-V5 production. Lanes: 1, no inducer (negative control); 2, IPTG for 3 h; 3, IPTG for 1 h; 4, co-induction with IPTG and arabinose for 3 h; 5, sequential induction (IPTG for 1 h, then arabinose for 3 h). (C) Liquid chromatography-tandem mass spectrometry analysis of peptides from excised gel bands (see Figure S6A). Identified peptides from three experimental replicates are shown. The table includes tryptic and non-tryptic peptides to detect cleavage events. Key: *PSM*, peptide-spectrum matches; *Coverage*, percentage of protein sequence covered. *Below the table.* Trx-PatXΔSS-MBP: Negative control (no Alr1666-V5). Trx-PatXΔSS-MBP + Alr1666-V5: Experimental assay with Alr1666-V5. ∗0 values: No non-tryptic peptides were detected in control samples.

Figure 5A shows the predicted molecular weights of the TrxA-PatXΔSS-MBP fusion protein in its uncleaved (∼63 kDa) and cleaved (∼18 kDa) forms. Consistent with this, we detected the full-length PatX fusion protein even in uninduced cultures (Figure 5B), likely due to promoter leakage. When alr1666 was expressed, a faint but reproducible band corresponding to the ∼18 kDa cleaved product appeared (Figure 5B); this band was absent in controls lacking Alr1666. Other bands—presumably TrxA-PatXΔSS-MBP degradation products—were observed under all conditions but did not correlate with alr1666 expression. Results were consistent across three biological replicates (Figure S5A). To test the specificity of this processing, we examined All2556, but detected no ∼18 kDa cleavage product (Figures S5B and S5C), underscoring Alr1666 as the cognate peptidase for PatX maturation.

The 18–25 kDa region was excised to identify PatX-cleavage peptides by mass spectrometry (Figure S6A). Because higher molecular-weight bands smear downward during SDS-PAGE, the excised slice also contains proteins from those upper regions, and they will be detected as well. We specifically screened for non-tryptic peptides that would indicate Alr1666-mediated processing. Two peptides met our stringent criteria: (1) their position corresponds to a cleavage in the PatX protein (2). They were uniquely detected in Alr1666-expressing samples (Figure 5B). These two peptides (AFSPSNSQNMISLK and GPDIIFWAHDR) flanked the HRGTGR inhibitory peptide and were exclusively present in peptidase-producing strains (Figures 5C and S6B), providing strong evidence that Alr1666 generated these cleavage products.

Collectively, our biochemical and genetic evidence identifies Alr1666 as the principal protease governing PatX activity, prompting its designation as PatP (PatX Peptidase).

### Phylogenomic co-evolution of *patX*-*hetR-patP* defines an Oscillatoriales-Nostocales clade and reveals an ancestral *patP* preceding *p*atX and *h*etR

We combined a broad genomic survey of *patP*, *hetR*, and *patX* with the established cyanobacterial phylogeny^34^ to trace how these genes have evolved across the phylum. The alr2656 gene was included in our analysis to further evaluate the PatP-PatX relationship.

Based on functional domains and SEC/SPI signal sequences, we identified 92, 541, 237, and 159 homologs of All2656, PatP, HetR, and PatX, respectively, across 474 cyanobacterial genomes covering all the taxonomic clades of this bacterial group (Table S3). All2656 showed restricted distribution, appearing in only 72 genomes (15.18%), with particularly high copy numbers in *Gloeobacter kilaueensis* JS1, *Leptolyngbya* sp. 7M, and *Mastigocoleus testarum* BC008, and phylogenetic analysis revealed its presence was limited to specific cyanobacterial orders, with 40 homologs found in Synechococcales—a pattern consistent with origin in this order followed by horizontal gene transfer to others (Table S3).

**Figure 6:**
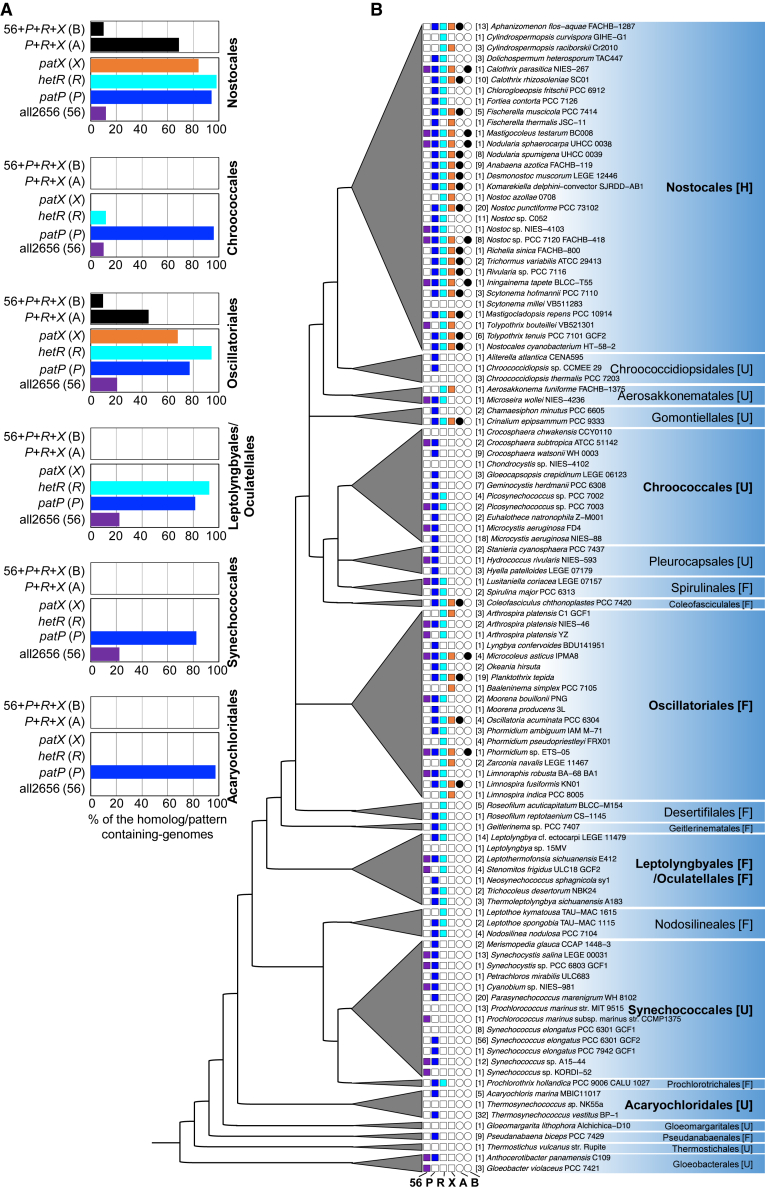
Phylogenetic distribution and co-evolution of genes in the HetR regulatory network (A) Prevalence of gene homologs and co-occurrence patterns across major cyanobacterial orders. (Bottom) Percentage of genomes containing homologs of *all2656* (violet), *patP* (blue), *hetR* (cyan), and *patX* (orange). (Top) Prevalence of two defined co-occurrence patterns in major orders: pattern A (P + R + X = *patP* + *hetR* + *patX*) and pattern B (56 + P +R + X = *all2656* + *patP* + *hetR* + *patX*). (B) Phylogenetic tree (adapted from^35^) and gene co-occurrence matrix. The left panel shows the cyanobacterial phylogeny with monophyletic orders represented by gray triangles. The right panel shows gene presence (filled squares) or absence (open squares) for each taxon, color coded as in A. Organisms are classified by order and family. Bracketed numbers indicate identical gene patterns within a family. The rightmost columns indicate the presence (●) or absence (○) of the two co-evolution patterns defined in (A). Cellular morphology is indicated: U, unicellular; F, filamentous; H, filamentous with heterocysts.

Striking co-evolutionary patterns between the *patP-patX-hetR* components were observed. While *patP* showed near-ubiquitous occurrence (86.2% of genomes), *patX* and *hetR* exhibited restricted but overlapping distributions, predominantly co-occurring in the filamentous orders Nostocales (63.5% and 49.7% of homologs, respectively) and Oscillatoriales (32.7% and 21.5%), suggesting functional linkage between these components (Figure 6A).

Analysis of gene presence (1) and absence (0) (ordered as all2656, *patP*, *hetR*, and *patX*) identified 14 distinct patterns, with three predominating: 0100 (37.5%; e.g., *Microcystis aeruginosa* NIES-88), 0111 (23.2%; e.g., *Trichormus variabilis* ATCC 29413), and 0110 (12.0%; e.g., *Leptolyngbya* cf. *ectocarpi* LEGE 11479) (Figure 6B). Notably, patterns 0111 (pattern A) and 1111 (pattern B)—associating the PatP peptidase with HetR and PatX—were restricted to (Nostocales, Gomontiellales, Spirulinales, and Oscillatoriales) and (Nostocales and Oscillatoriales), respectively. These results demonstrate the co-evolution of *patP*, *hetR*, and *patX* primarily within these clades, strongly supporting the functional integration of PatP-mediated PatX processing with HetR regulation in these lineages (Figure 6B).

In contrast, the predominant 0100 pattern (37.5% of genomes), where only *patP* is present, was characteristic of unicellular strains like *Microcystis aeruginosa*, consistent with PatP’s ancestral role in other cellular processes before its recruitment for filament-growth control and heterocyst patterning systems (Figure 6B).

These data establish PatP as an ancestral, cyanobacteria-wide peptidase whose later, clade-restricted coupling with PatX and HetR created the regulatory module that underpins heterocyst patterning.

## Discussion

Multicellularity has evolved repeatedly across the Tree of Life,^36^^,^^37^ yet in most lineages, the genetic innovations that converted simple cellular consortia into organisms with reproducible spatial patterns remain poorly understood. Multicellular cyanobacteria offer a uniquely tractable model: their differentiation of heterocysts from vegetative cells is governed by a small set of lineage-specific regulators that arose on a genomic background shared with unicellular relatives.^38^^,^^39^ We show here that the patterning peptide PatX is matured by a peptidase, PatP, that was likely already present in the last common ancestor of all cyanobacteria (Figure 6). This finding reveals how an ancient proteolytic function was co-opted to couple intercellular communication to developmental fate, providing a mechanistic link between the emergence of multicellularity and the evolution of cell-type diversity.

The PatX peptide harbors the canonical HRGTGR motif that docks into HetR^15^ and neutralizes its DNA-binding activity (Figures 1A and 1B), yet its biogenesis is fundamentally different from that of the two other HetR antagonists. Unlike PatS and HetN, whose precursors are likely translated and cleaved in the cytoplasm, PatX is equipped with a Sec-dependent signal peptide that directs it outside the cytosol (Figures 1D and 1E). Once there, PatX is matured by the broadly conserved peptidase PatP (Figures 3, 4A, and 5B). A cleavage event at 27 residues N-terminal to the HRGTGR motif was identified in the *E. coli-*based reporter (Figures 5B and S6B). If the site is conserved in *Nostoc*, PatX would undergo processive maturation analogous to PatS.^40^ Processive proteolysis is well-established; Lon protease, an ATP-dependent AAA+ enzyme conserved across all three life kingdoms, maintains proteostasis by degrading misfolded proteins and modulates stress responses, virulence, and bacterial group behavior through selective turnover of regulatory proteins.^41^

Extracytosolic export is required for PatP and PatX activity, so the decisive question is which compartment they enter. In cyanobacteria whose thylakoid and plasma membranes are distinct (e.g., *Nostoc*), the two membranes maintain functionally separate Sec systems.^31^ An *E. coli* Tat reporter peptide directs GFP exclusively to the periplasm, with no lumenal signal detected,^42^ and dedicated type-I signal peptidases for each membrane reinforce this specificity.^43^ Moreover, *E. coli* SecA accepts only periplasm-targeting peptides and ignores thylakoid-lumen motifs.^30^ Because the PatP and PatX signal peptides drive efficient Sec-dependent export of MBP to the *E. coli* periplasm, they must themselves be periplasm-targeting. This conclusion is corroborated by the polar peripheral fluorescence of the PatP-sfGFP fusion in *Nostoc* (Figure 4B), by the periplasmic-type features of PatP signal sequence, and by the observation that a PatP homolog in *Synechococcus* sp PCC 7002 is located in the periplasm.^31^

**Figure 7:**
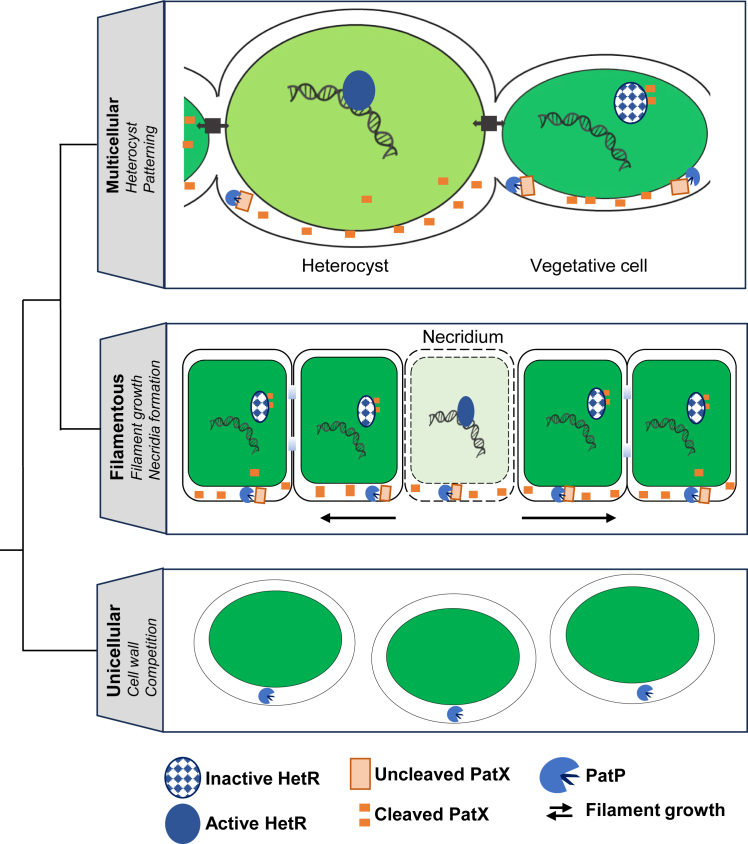
Model for the evolutionary trajectory of the PatP peptidase and its co-option by the PatX signaling peptide The putative peptidase PatP (PatX peptidase) is ancestral among cyanobacteria. In unicellular species, it may process peptides involved in cell wall remodeling, niche competition, or amino acid acquisition (in italics). *patX-hetR* genes emerged later in filamentous cyanobacteria (e.g., Oscillatoriales). PatP would be co-opted to process the novel signaling peptide PatX, creating a new system to regulate filament growth, likely via necridia formation, in response to metabolic or environmental cues (in italics). Localized cell lysis at a necridium (light cell) results in filament fragmentation and the release of two independent filaments. In heterocyst-forming cyanobacteria (e.g., *Nostoc*), this PatP-PatX system was further specialized to control heterocyst pattern formation. Initiation: Combined nitrogen starvation triggers of *hetR* and *patX* transcription. Processing: PatX is exported to the periplasm and cleaved by PatP to generate an active signal (e.g., a hexapeptide, orange dots). Patterning: The PatX-derived signal (orange dots) diffuses along the filament, inhibiting the master regulator HetR to space developing heterocysts. Protection: In pro-heterocysts, HetL binds HetR, shielding it from inhibition and allowing differentiation. In the heterocyst, HetR interacts with its target genes. In the vegetative cells, HetR is bound to PatX-6 and cannot interact with the DNA. Succession: In strains with additional systems, the early PatX signal is later superseded by PatS and HetN-derived signals (not shown in the figure).

*patX* transcripts accumulate specifically in prospective heterocysts,^23^ whereas *patP* is expressed along the filament. PatP production within heterocysts cannot be excluded, yet vegetative cells—whose numerical dominance along the filament guarantees a far larger cumulative supply of the protease—are expected to provide the bulk of the activity. Consequently, PatX precursor synthesized in the heterocyst can be exported and converted to the active peptide by PatP contributed by surrounding vegetative cells. This two-cell interplay—precursor origin in the heterocyst, maturation by protease from adjacent vegetative cells—allows local PatP activity and subsequent peptide diffusion to jointly establish the periodic pattern **(**Figure 7).

Our comparative genomic analysis retraces the evolutionary assembly of the PatP-HetR-PatX regulatory module, revealing a gradual transition from an ancestral protease to a specialized developmental circuit (Figures 6 and 7). The widespread presence of *patP* across cyanobacterial genomes, including those of numerous unicellular strains, suggests an early role, possibly linked to cell wall remodeling, stress adaptation, or competitive interactions within microbial niches (Figure 7). This ancestral versatility is consistent with its presence across diverse lineages and its subsequent association with regulatory networks in filamentous branches.

The co-occurrence of *patP*, *hetR*, and *patX* in both Oscillatoriales and Nostocales indicates that this association arose prior to the emergence of heterocysts. These lineages subsequently diverged: Oscillatoriales, which do not form heterocysts, retained HetR homologs likely serving alternative regulatory roles, such as the control of filament growth, whereas Nostocales co-opted the ancestral module to regulate heterocyst differentiation and spatial patterning (Figure 7). Mapping PatP activity in unicellular and filamentous cyanobacteria will pinpoint the ancestral proteolytic event that was later co-opted as the multicellular patterning signal.

This evolutionary trajectory exemplifies functional co-option, in which a pre-existing stress- or filament-related regulatory network was repurposed to drive cellular specialization. The emergence of the PatP–PatX–HetR circuit in Nostocales thus represents a key evolutionary innovation—transforming a generic regulatory module into the developmental core of heterocyst formation and supporting the rise of complex multicellularity in cyanobacteria. Such co-option mirrors the evolutionary trajectories found in metazoan signaling pathways: In the Notch signaling system, ligands produced by signal-sending cells are distributed on the cell membrane and bind to NOTCH receptors on signal-receiving cells. This triggers a proteolytic cascade releasing the Notch intracellular domain, which enters the nucleus to co-activate transcription.^44^ In BMP signaling (Bone Morphogenetic Protein), Tolloid metalloproteinases cleave the secreted antagonist Chordin/Sog, releasing BMP inhibition and generating a morphogen gradient that patterns embryonic tissues.^45^ Thus, cyanobacteria, like metazoans, have repeatedly solved the multicellularity problem by repurposing ancestral proteases—PatP for PatX, ADAM/Tolloid for Notch/BMP—to cleave lineage-specific ligands and create spatially resolved developmental instructions.

### Limitations of the study

While our data establish a model for PatX maturation by the conserved peptidase PatP, this study has limitations that point to future directions. The proposed processive cleavage of PatX was identified using an *E. coli* reporter system; confirming the precise cleavage sites and the processivity of this maturation *in vivo* within *Nostoc* remains to be accomplished. Furthermore, although our localization data strongly suggest PatP and PatX function in the periplasm, direct biochemical evidence verifying the exact compartment where their interaction occurs in cyanobacteria is still needed. Finally, the native substrate and physiological role of PatP in unicellular cyanobacterial relatives and in Oscillatoriales, which is key to understanding the evolutionary precursor to this patterning system, remain to be identified.

## Resource availability

### Lead contact

Further information and questions or inquiries about data and resources should be directed to and will be fulfilled by the lead contact, Amel Latifi (latifi@imm.cnrs.fr).

### Materials availability

All the mutant strains generated in this study will be shared upon request to the lead contact.

### Data and code availability

• All the data generated in this study are presented within the main/supplementary figures and tables.
• No code was generated or used in this study.
• Any additional information is available from the lead contact upon request.

## Acknowledgments

The authors thank Régine Brun from the proteomic platform (CNRS, IMM) for mass spectrometry analyses, Dr. Laetitia Pieulle for providing the anti-thioredoxin (TrxA), Dr. Bérengère Ize for helpful discussion regarding SEC-dependent export, and Dr. Véronique Risoul for technical assistance. The project was supported by the French agency “Agence Nationale pour la Recherche Scientifique” (ANR-21-CE20-0025-01) and by funding from Aix Marseille University. Xu Xiaomei was funded by a fellowship from the Chinese Government.

## Author contributions

A.L. conceptualized the study and obtained the funding; X.X., A.S., S.C., J.D., and Z.Y. were responsible for the physiological and genetic analysis; E.T. performed the phylogenomics analysis; Y.D. supervised the quantitative transcription analysis, and D.B. supervised the ITC analysis; M.B. and B.D. contributed to experiment design and data analysis; A.L. wrote the manuscript. All authors participated in data interpretation and manuscript editing.

## Declaration of interests

The authors declare no competing interests.

## STAR★Methods

### Key resources table

**Table:** 

REAGENT or RESOURCE	SOURCE	IDENTIFIER
**Antibodies**		

V5 tag antibodies (SV5-Pk1)	Thermo Fisher	Cat# R960-25; RRID:AB_2556564
TrxA antibodies	L. Pieulle	N/A

**Bacterial strains**		

*Nostoc* PCC 7120	LCB substrain, originated for Pasteur Institute Cyanobacteria Collection.	PCC 7120
*E. coli* K-12 MG1655	Keo collection	MG1655
*E. coli* MG1655 *ΔmalE*	Keo collection	*malE* mutant
*E. coli* BTH101	Euromodex	BTH101

**Chemicals, peptides, and recombinant proteins**		

In-Fusion® Snap Assembly Master Mix	Takara	638948
CloneAmp HiFi PCR premix	Takara	639298
PatX-6 peptide (HRGTGR)	Genecust	N/A

**Experimental models: Organisms/strains**		

See Table S4	N/A	N/A

**Oligonucleotides**		

See Table S6	N/A	N/A

**Recombinant DNA**		

See Table S5	N/A	N/A

**Software and algorithms**		

PEAQ-ITC	Malvern Panalytical	https://www.malvernpanalytical.com/en/products/product-range/microcal-range/microcal-itc-range/microcal-peaq-itc
SignalP-5.0	DTU Health Tech	https://services.healthtech.dtu.dk/services/SignalP-5.0/
DeepTMHMM	Biolib	https://dtu.biolib.com/DeepTMHMM
ImageJ	NIH	https://imagej.net/ij/

### Method details

#### Growth conditions, conjugation, and heterocyst induction

*Nostoc* and derivatives were grown in BG11 medium at 30 °C under continuous illumination (40 μE m^−2^s^−1^). Concentrations of antibiotics, the induction of heterocysts in media lacking a source of combined nitrogen, and conditions for microscopy were described previously.^24^ To evaluate its effect on heterocyst formation, PatX6 peptide (synthesized by Genecust: https://www.genecust.com/en/) was added to the culture to a final concentration of 1 μM. Conjugation of *Nostoc* was performed as described by Cai & Wolk,^46^ with modifications in.^24^

To monitor cellular differentiation, heterocyst formation was tracked in two complementary ways: (i) bright-field microscopy, which exploits the distinct morphology of heterocysts, and (ii) fluorescence microscopy, taking advantage of the red autofluorescence emitted by vegetative cells due to their photosynthetic pigments. Because oxygenic photosynthesis is absent in heterocysts, these cells appear dark against the fluorescent filaments.

#### Microscopy

Microscopic observations were conducted using a Nikon Eclipse E800 optical microscope with a ×100 magnifying objective. Images were captured with a Nikon DXM 1200 digital camera, equipped with a PE-300lite (CoolLED) light source and controlled via ATC-1 software. Bright field observations used white light, GFP fluorescence used a FITC filter (Ex 465/495 nm, Dm 505 nm), and photosynthetic pigment fluorescence used a TRITC filter (Ex 540/25, Dm 565). We standardized fluorescence images by acquiring them under identical settings (including exposure time) and subtracting the background signal using a wild-type strain that does not produce the fluorophore.

#### Isothermal titration calorimetry (ITC)

ITC was performed as described in.^24^ Briefly: reactions were carried out at 25 °C in PBS pH 7.4 (chosen to avoid buffer mismatch) using a MicroCal PEAQ-ITC (Malvern Panalytical). The protein ligand (cell, 200 μL) was titrated with the peptide analyte (syringe) via 19 injections: an initial 0.4 μL shot followed by 18 aliquots of 2 μL delivered at 4 s per injection with 150 s intervals between them; the stirrer was kept at 750 rpm. A constant heat offset determined from the pre- and post-injection baselines was subtracted to correct for dilution heat, and the integrated heats were fitted to a one-site binding model with the PEAQ-ITC Analysis Software. The experiment was repeated three times with independent protein purifications, and one representative result is shown.

The association constant K_a_ (and its reciprocal, the dissociation constant Kd) is obtained directly from the fitted binding isotherm as the concentration-independent parameter that describes the ligand–analyte interaction.

#### Maltose-binding protein (MBP) localization assay

*E. coli* K-12 MG1655 strains were cultured overnight in LB medium at 30 °C, then plated on MacConkey agar with 0.4% maltose and 1 mM IPTG. After two days at 30 °C, strains with periplasmic MBP (using maltose) showed red colonies, while strains with cytosolic MBP appeared white.

#### Cleavage assay

*E. coli* strains producing TrxA-PatX-MBP fusion and Alr1666 were lysed, and proteins were separated by SDS-PAGE on 12% polyacrylamide gels. The gels were either stained with Coomassie blue or electroblotted onto Immobilon membranes. Immunoblot analysis was carried out either with anti-V5 (Thermo Fisher, diluted 1/2000) or anti-TrxA (from Dr. L. Pieulle, diluted 1/5000). For protein identification, following Coomassie blue staining, bands corresponding to the molecular mass of cleaved PatX were excised and analyzed using LC-MS/MS as described in ref.^47^, with a minor modification to include the identification of both trypsin and non-trypsin-generated peptides.

#### Bioinformatic analysis

The signal sequences of the studied proteins were analyzed using SignalP-5.0^48^ (version available in 2022).

For comparative genomics analysis, the genome data of 474 cyanobacterial strains available in March 2025 were downloaded from NCBI: https://ftp.ncbi.nlm.nih.gov/ (Table S3). This dataset includes all complete genomes at the highest assembly level, their RefSeq category (reference or representative genome, where available), as well as their annotation features and taxonomic lineages. The ability of strains to form heterocysts was obtained from the literature.^35^^,^^49^^,^^50^^,^^51^ Hidden Markov Models (HMMs) of protein family profiles (Pfam (version 37.2, March 2025) and Pgap (version 17, March 2025)) were downloaded from ftp.ebi.ac.uk/pub/databases/Pfam/releases/ and ftp.ncbi.nih.gov/hmm/current/ftp sites, respectively. Reference seed proteins from *Nostoc* (retrieved from the UniProt database: www.uniprot.org) are listed in Table S3.

The HMMER package^52^ and HMM domain profiles were used to identify seed functional domains associated with reference proteins (Table S3). Alignments scoring above the trusted cutoffs were considered significant.^52^

Since PatX is small, we also searched for it in pseudogenes and intergenic regions. These regions (excluding known genetic elements) were extracted, translated in six frames using getorf (∗-minsize 30∗, bacterial codon usage, methionine start codons), and merged with proteomes for analysis. Next, HMMER3^52^ and custom Perl scripts screened complete genomes (including pseudogenes and intergenic-derived proteins) for homologs containing reference seed domains. Putative homologs required ≥1 seed domain (Table S3), with alignments above trusted cutoffs deemed significant. Putative homologs were further analyzed for additional functional domains using the same tools, and domain architectures were defined via in-house Perl scripts. Pre-homologs were selected based on strict seed domain presence (correct order, no extra domains). Finally, All2656, Alr1666, and PatX pre-homologs were screened for the presence of SEC/SPI signal peptides (SignalP 6.0, slow mode^53^) and absence of transmembrane domains (DeepTMHMM^54^).

#### Bacterial two-hybrid assays

Bacterial two-hybrid assays followed the method of Karimova et al. (1998).^1^ The BTH101 strain, co-transformed with T18- and T25-fusion plasmids, was plated on LB containing ampicillin and kanamycin and incubated at 30°C for two days. For each assay, 10 colonies were grown in 3 ml of LB with antibiotics, and 0.5 mM IPTG overnight at 30°C. ß-Galactosidase activity, measured according to,^2^ is reported as means from three independent assays.

#### Electrophoretic mobility shift assays (EMSA)

The *hetP* gene promoter (alr2818) was amplified by PCR using *PhetP* fw and *PhetP* rv primers, with the forward primer labeled at the 5′ end with 6-FAM. Purified HetR protein (as previously described),^3^ was incubated with the *hetP* promoter (50 nM) in a buffer containing 10 mM Tris (pH 8), 150 mM KCl, 500 nM EDTA, 0.1% Triton X-100, 12.5% glycerol, 1 mM DTT, and 1 μg DiDC competitor at 4°C in the dark for 30 minutes. Electrophoresis was conducted at 250 V for 60 minutes, and DNA was visualized using the Typhoon FLA 9500. The experiment was repeated with independent protein purifications, and one representative result is shown.

#### RNA preparation, reverse transcription, and quantitative real-time PCR

RNAs were prepared from *Nostoc* cultures (15 ml, OD750 = 1) using the Qiagen RNA extraction kit per manufacturer’s instructions, with an additional TURBO DNase (Invitrogen) digestion to remove DNA contamination. RNA quality was assessed with an Agilent tape station and quantified at 260 nm using a NanoDrop 1000 (Thermo Fisher). For cDNA synthesis, 1 μg of total RNA and 0.5 μg of random primers (Promega) were used with GoScript™ Reverse Transcriptase (Promega) as per instructions. Quantitative real-time PCR (qPCR) was conducted as previously described.^3^

#### Synthetic peptide or DNA fragments

DNA fragments (*patXXSS1*, *patXXSS2*, all2656*SS* and alr1666*SS*) were synthesized by Eurofins :https://eurofinsgenomics.eu/en/eurofins-genomics-genomic-services-by-experts/.

#### Mutant construction

All mutants were created using the CRISPR-Cpf1 system present on the pMB39 plasmid. For each gene, we amplified 1 kbp upstream and downstream regions from *Nostoc* gDNA using RP-up-Fwd/Rev and RP-down-Fwd/Rev primers, respectively. These fragments were then cloned into pMB39 between the BglII and PstI sites using In-fusion. We designed spacer sequences as described in.^4^ Gene-specific Spacers -Fwd and Rev primers were annealed at 95°C to generate spacers specific to each gene, which were then ligated into the AarI sites of the pMB-RP plasmid. We introduced the editing plasmids into *Nostoc* via conjugation.

Exconjugants were isolated on BG11 plates supplemented with 2.5 μg/mL spectinomycin and streptomycin for eight successive generations, then grown in liquid culture for gDNA extraction. We confirmed the absence of the edited gene and complete segregation of the mutants by PCR on gDNA. Fully segregated clones were plated on BG11 plates without antibiotics and then on plates with 10% sucrose to counter-select the pMB editing plasmid. We verified the loss of the editing plasmid by colony PCR, plating the resulting strain on BG11 supplemented with 2.5 μg/mL spectinomycin and streptomycin. All experiments were conducted on two independent clones.

#### Plasmid construction

The strategy used for plasmid construction is briefly described below. All the constructs that utilized primers designed as “infusion primers” were obtained using the Takara In-Fusion® HD Cloning Kit. All the recombinant plasmids obtained were analyzed by sequencing. In silico plasmid maps are available upon request.

pKT25-*patXΔSS*: the open reading frame of *patXΔSS*, where *patX* lacks the first 66 nucleotides (encoding the putative signal sequence (SS)), was amplified using the *patXΔSS* dh fw T25 and *patXΔSS* dh rv primers and cloned into the PstI and EcoRI restriction sites of the pKT25 plasmid.

pUT18C-*patXΔSS*: the open reading frame of *patXΔSS* was amplified using the *patXΔSS* dh fw T18 and *patXΔSS* dh rv primers and cloned into the PstI and EcoRI restriction sites of the pUT18C plasmid.

*patXΔSS*-pKNT25: the open reading frame of *patXΔSS* was amplified using the *patXΔSS* dh fw T18C or NT25/T18 and *patXΔSS* dh rv NT25/T18 primers and cloned into the PstI and EcoRI restriction sites of the pKNT25 plasmid.

*patXΔSS*-pUT18: the open reading frame of *patXΔSS* was amplified using the *patXΔSS* dh fw T18C or NT25/T18 and *patXΔSS* dh rv NT25/T18 primers and cloned into the PstI and EcoRI restriction sites of the pUT25 plasmid.

pRL1272-P*petE-patX*: the open reading frame of *patX* with an extra 500 bp after the stop codon was amplified using the *patX* pRL fw and *patX* pRL rv infusion primers and cloned into the BamHI restriction site of the pRL1272-P*petE* replicative plasmid in *Nostoc*.

pRL1272-P*petE-patXΔSS*: the open reading frame of *patXΔSS* with an extra 500 bp after the stop codon was amplified using the *patXΔSS* pRL fw and *patX* pRL rv infusion primers and cloned into the BamHI restriction site of the pRL1272-P*petE* replicative plasmid in *Nostoc*.

pRL25T-P*petE-*alr1666ΔSS: the open reading frame of alr1666ΔSS (where alr1666 lacks the first 84 nucleotides (encoding the putative SS)) was amplified using the alr1666*ΔSS* pRL fw and alr1666 pRL rv infusion primers and cloned into the BamHI restriction site of the pRL25T-P*petE* replicative plasmid in *Nostoc*.

p33tac-*malE*: The open reading frame of *malE* was amplified using the *malE* fw and *malE* rv infusion primers and cloned between the XbaI and HindIII restriction sites of the p33tac expression plasmid.

p33tac-*malEΔSS*: the open reading frame of *malEΔSS,* where *malE* lacks the first 78 nucleotides, which encode the SS, was amplified using the *malEΔSS* fw and *malEΔSS* rv infusion primers and cloned into the XbaI and HindIII restriction sites of the p33tac expression plasmid.

p33tac-*patXSS1*-*malEΔSS*: the DNA fragment of *patXXSS1,* which includes the first 99 nucleotides (encoding the putative SS) and the hydrophobic domain, was cloned into the NdeI restriction site of the p33tac-*malEΔSS* expression plasmid.

p33tac-*patXSS2*-*malEΔSS*: the DNA fragment of *patXXSS2,* which includes the first 72 nucleotides encoding the putative SS, was cloned into the NdeI restriction site of the p33tac-*malEΔSS* expression plasmid.

p33tac-alr1666*SS*-*malEΔSS*: the DNA fragment of alr1666*SS,* which includes the first 87 nucleotides encoding the putative SS, was cloned into the NdeI restriction site of the p33tac-*malEΔSS* expression plasmid.

pMB38 (pSL2680-*cpf1*): The *cpf1* gene under the *lac* promoter and the *Francisella novicida* CRISPR array were PCR-amplified from pSL2680^55^ using primer pairs OMB87/OMB88 and OMB89/OMB90, respectively. The resulting fragments were then cloned into BamHI/BglII-digested pRR001.^56^

pMB39 (pMB38-*sacB*): The s*acB* coding sequence and its promoter were amplified from pRL271^46^ using primers OMB93/OMB94 and cloned into BamHI-linearized pMB38 plasmid.

p33tac-*patX*: a fusion between *trxA-patX*Δ*SS-malE* was obtained by Infusion and cloned into XbaI and HindIII restriction sites of the p33tac plasmid. For this, a fragment including the trxA and *patX*Δ*SS* was amplified from the pLIC-PatX plasmid using the *TrxAPatXMBP ptac* 1 fw and *TrxPatXMBP ptac* 2 rv primers. The *malE* open reading frame was amplified from *E. coli* genomic DNA using the *TrxAPatXMBP ptac* 3 fw and *TrxAPatXMBP ptac 4* rv. The two obtained fragments were then cloned into the p33tac plasmid, digested with XbaI and HindIII.

pBAD-alr1666ΔSS-V5: the open reading frame of alr1666ΔSS (where alr1666 lacks the first 84 nucleotides (encoding the putative SS)) fused with the V5 tag was amplified using the alr1666-V5 fw/rv primers and cloned into the pBAD plasmid, digested with EcoRI and XbaI. pBAD-all2656ΔSS -V5: the open reading frame of all2656ΔSS (where all2656 lacks the first 75 nucleotides (encoding the putative SS)) fused with the V5 tag was amplified using the all2656-V5 fw/rv primers and cloned into the pBAD plasmid, digested with EcoRI and XbaI.

## Supplemental information

Data S1. Supplemental figures and tables

Table S3. Genomics analysis Sheet 1. List of protein seeds used in this study. Sheet 2. List of cyanobacterial genomes analyzed with their features and the number of homologs from each protein seed. Sheet 3. List of protein homologs identified. Sheet 4. Distribution of protein homologs across taxonomic orders. Sheet 5. Distribution of protein homologs across taxonomic families.

Table S6. Primers used in this study
