# Supplementary material for: Co-option of an ancestral peptidase controls developmental patterning in multicellular cyanobacteria
Source: iScience. 2025 Nov 28;29(1):114265. doi: 10.1016/j.isci.2025.114265 (PMC12756182; doi:10.1016/j.isci.2025.114265)
Supplement: Document S1. Figures S1–S6 and Tables S1, S2, S4, and S5 [file mmc1.pdf]

iScience, Volume *29*

Supplemental information

Co-option of an ancestral peptidase controls developmental patterning in multicellular cyanobacteria

Xiaomei Xu, Anaïs Scholivet, Stéphanie Champ, Matthieu Bergé, Zulihumaer Yeerkenjiang, Jonas Desjardins, Yann Denis, Badreddine Douzi, Deborah Byrne, Emmanuel Talla, and Amel Latifi

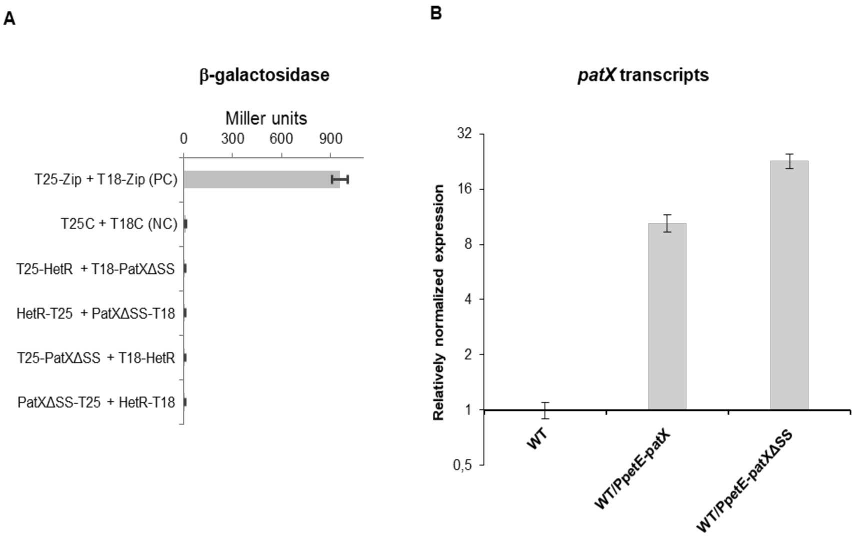

Figure S1. Analysis of PatX and HetR interaction and *patX* expression. (A) Bacterial two-hybrid assay between PatXΔSS and HetR. Data are represented as mean +/- SEM indicated by error bars. **(B)** qRT-PCR analysis of *patX* and *patXΔSS* expression relative to the experiment shown in Fig. 1*C*. Data are represented as mean +/- SEM indicated by error bars. The values obtained from WT were set to 1.

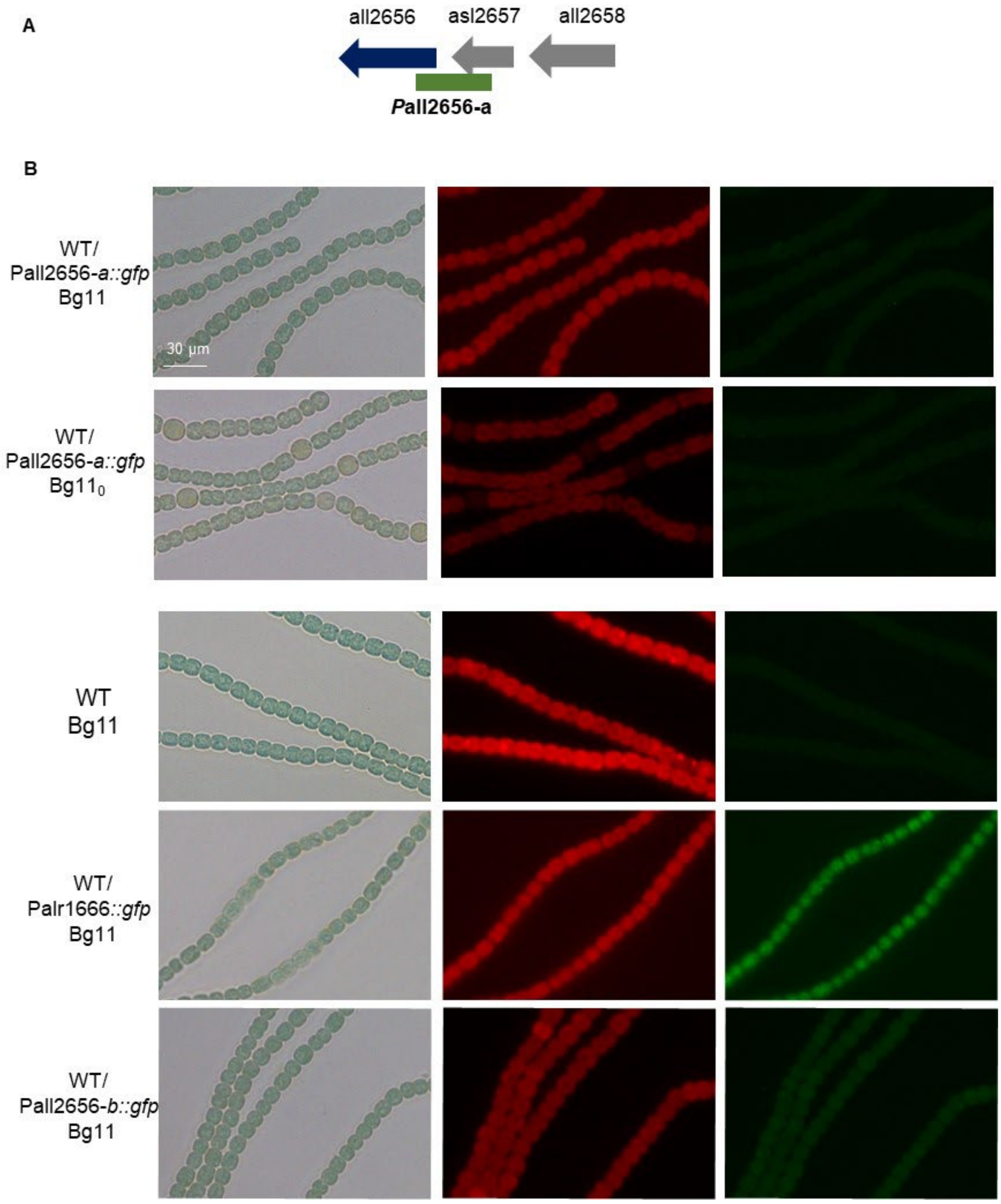

Figure S2. Genomic organization and expression of all2656 gene. (A) Genomic organization of all2656 gene. The segment in green is the gene part that was fused to GFP.

(B) Microscopic bright field images (left), auto-fluorescence images (middle), and GFP-fluorescence images (right) of indicated *Nostoc* strains grown in BG11 or 24 hours after transfer into $BG11_0$ medium. Scale bars: 30 µm.

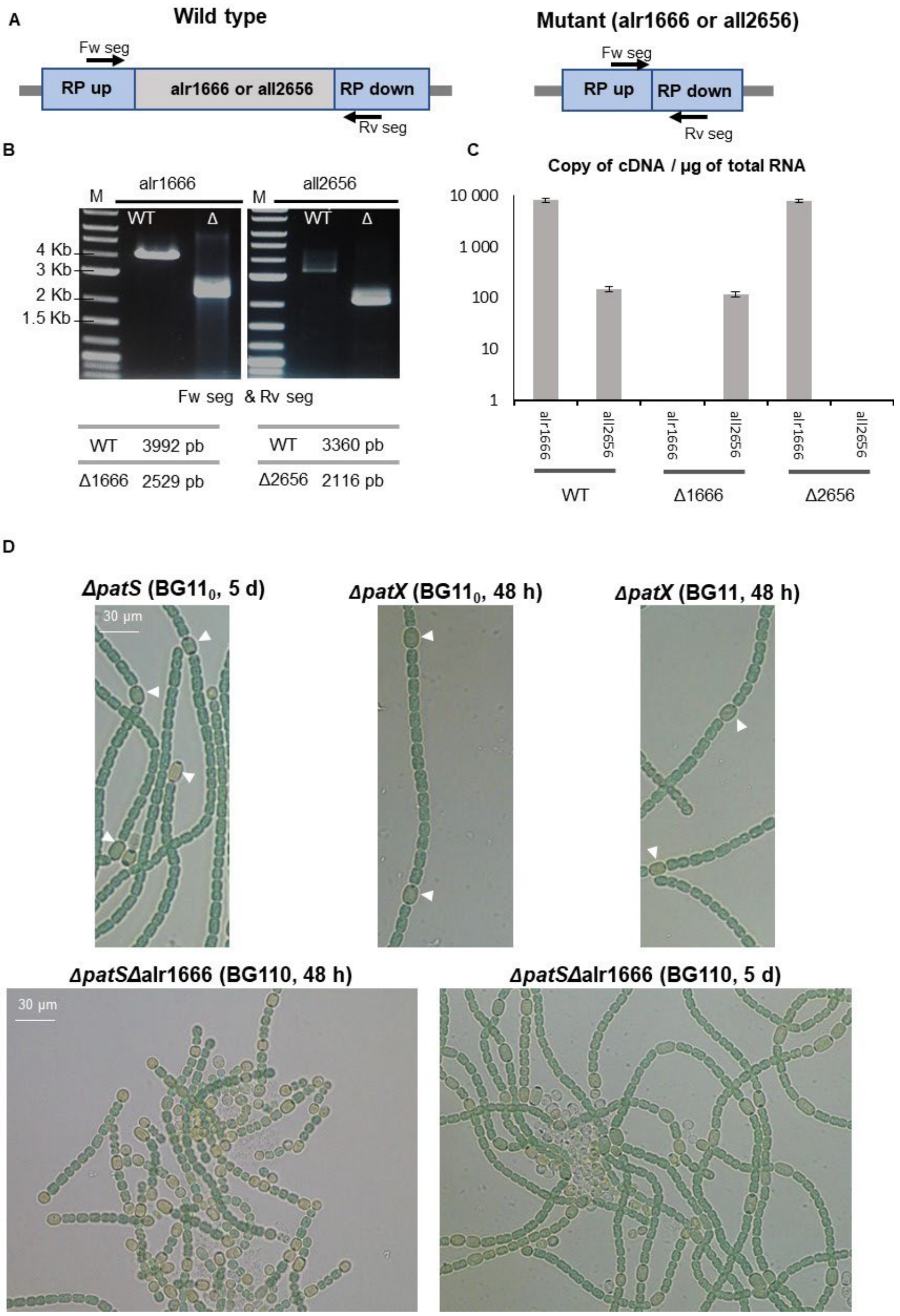

Figure S3. Generation and phenotype analysis of *Nostoc* mutant strains. (A) Schematic representation of the WT and mutant genomic region genes indicating the positions of the primers used to analyze the segregation. **(B)** PCR on genomic DNA of WT, *Δ*alr166*6,* and *Δ*all2656 strains. The expected sizes for the PCR product of each strain are indicated at the bottom. Δ indicates single mutants. **(C)** qRT-PCR analysis of the expression of alr1666 and all2656 genes in the indicated strains. The strains were grown in BG11. The data are presented as absolute quantification of the transcript. Data are represented as mean +/- SEM indicated by error bars. (**D**) Microscopic bright field images of indicated *Nostoc* strains 48h or 5 days after transfer from BG11 plates to BG110 liquid medium White arrows indicate heterocysts. Scale bars: 30 µm.

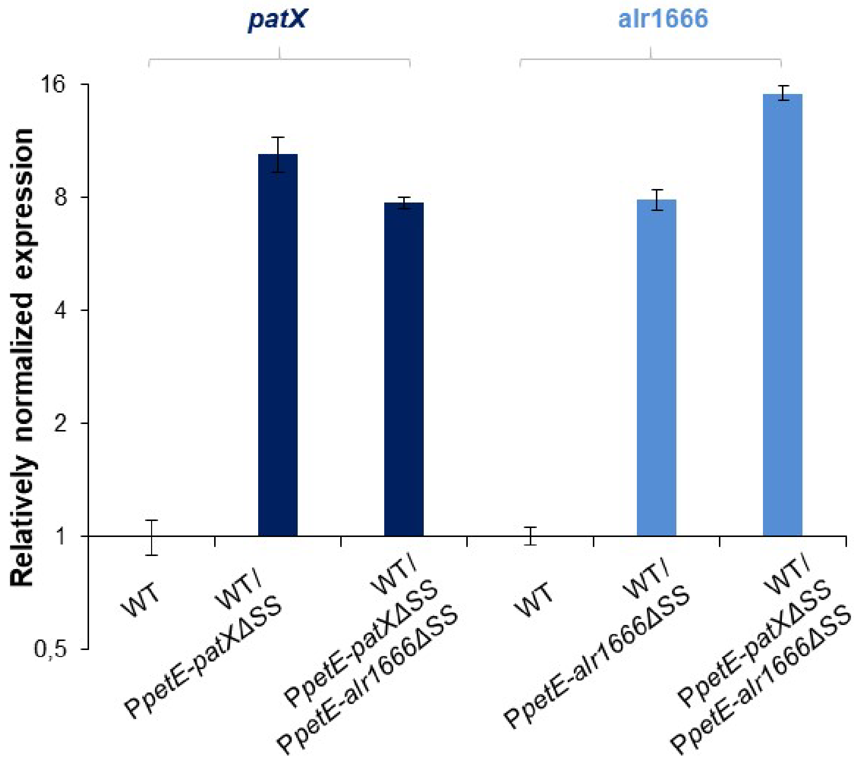

Figure S4. Expression analysis of *patXΔSS* and alr1666*ΔSS*. qRT-PCR analysis of *patX∆SS* and alr1666*∆SS* expression relative to the experiment shown in Fig. 4*A*. Data are represented as mean +/- SEM indicated by error bars. The values obtained from WT were set to 1.

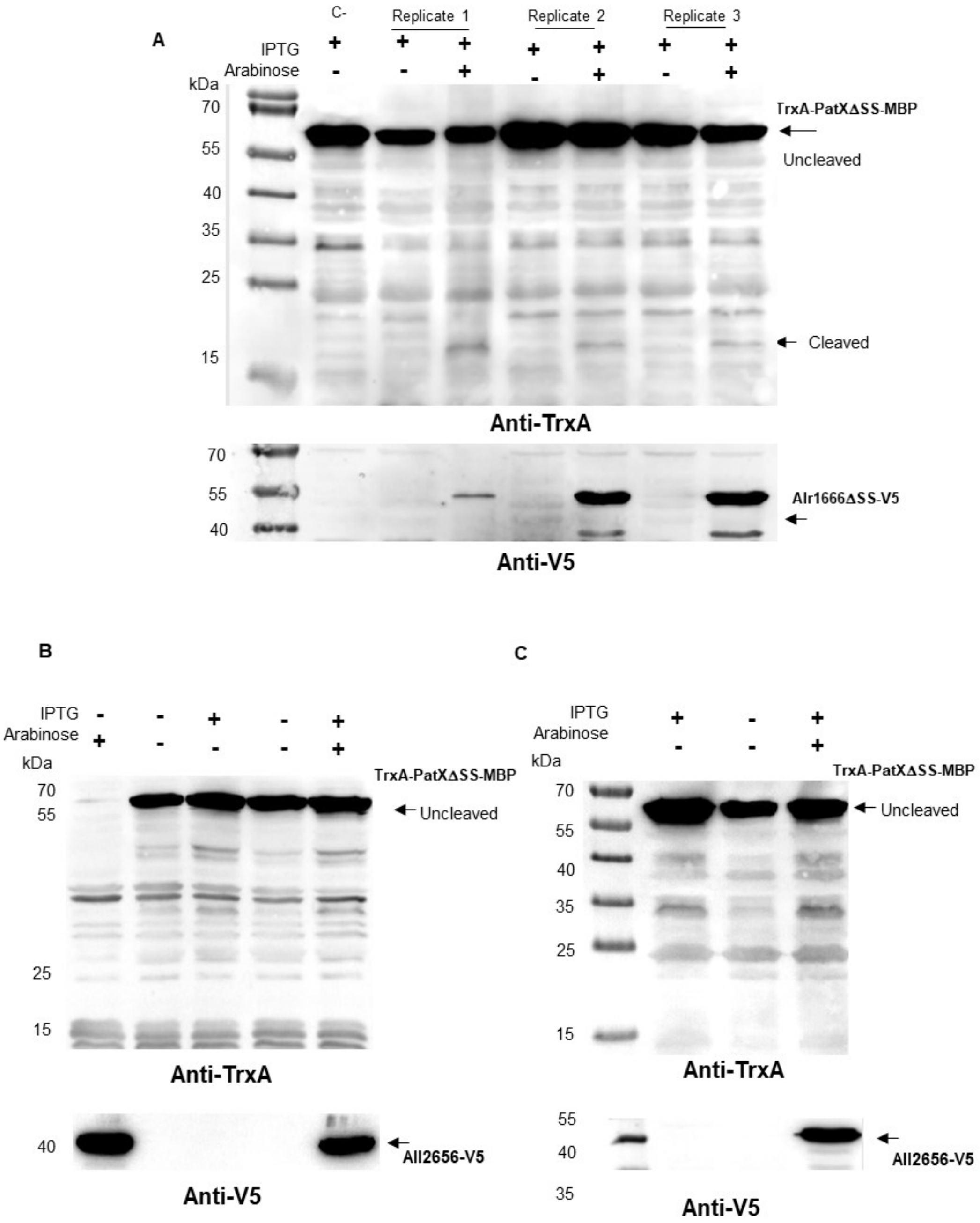

Figure S5. Proteolytic cleavage assay of PatX fusion protein. Cleavage assay of TrxA-PatXΔSS-MBP by Alr1666ΔSS **(A)**, or All2656ΔSS **(B, C).** IPTG induces the transcription of *trxA-patXΔSS-malE*. Arabinose induces the transcription of alr1666*ΔSS* -V5 or all2656*ΔSS* -V5. C: negative control (strain harboring the plasmid expressing the TrxA-PatXΔSS-MBP construct) Replicates: independent cultures of the strain expressing the TrxA-PatXΔSS-MBP and Alr1666-V5 constructs)

A

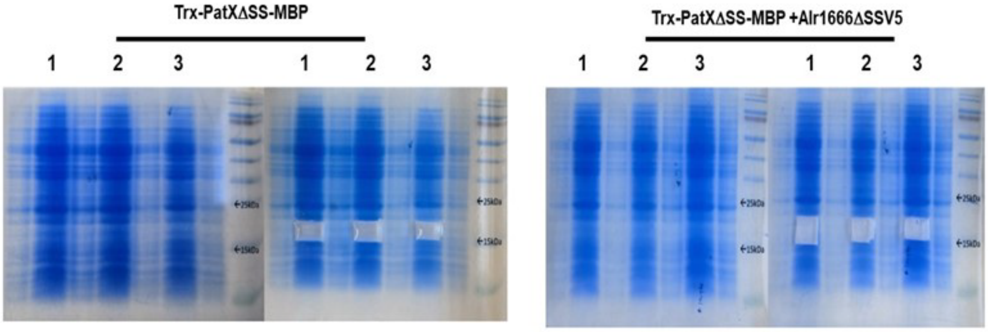

B

MGHHHHHHSDKIIHLTDDSFDTDVLKADGAILVDFWAEWCGPCK
MIAPILDEIADEYQGKLTVAKLNIDQNPGTAPKYGIRGIPTLLL
FKNGEVAATKVGALSKGQLKEFLDANLAGSGSGVSGVDLGTENL
YFQS**MLGIASHDFAFSPSNSQNMISLKSKPKRNQPEKPQPHRGT**
GRRSLIETYSNAHLIAYPYDVPDYAKIEEGKLVIWINGDKGYNG
LAEVGKKFEKDTGIKVTVEHPDKLEEKFPQVAATGDGPDIIFWA
HDRFGGYAQSGLLAEITPDKAFQDKLYPFTWDAVRYNGKLIAYP
IAVEALSLIYNKDLLPNPPKTWEEIPALDKELKAKGKSALMFNL
QEPYFTWPLIAADGGYAFKYENGKYDIKDVGVDNAGAKAGLTFL
VDLIKNKHMNADTDYSIAEAAFNKGETAMTINGPWAWSNIDTSK
VNYGVTVLPTFKGQPSKPFVGVLSAGINAASPNKELAKEFLENY
LLTDEGLEAVNKDKPLGAVALKSYEEELAKDPRIAATMENAQKG
EIMPNIPQMSAFWYAVRTAVINAASGRQTVDEALKDAQTRITK-

Figure S6. Analysis of Trx-PatXΔSS-MBP Cleavage. (**A**) SDS PAGE electrophoresis for mass spectrometry identification after cleavage assay. 1, 2, and 3 indicate crude extract proteins from three independent assays. **(B)** Amino acid sequence of the Trx-PatXΔSS-MBP fusion, with non-tryptic peptides identified by mass spectrometry (underlined). The PatXΔSS sequence is bolded, and the inhibitory peptide is highlighted in red.

**Strain**	**% of heterocysts, 48H after transfer**
patX over-expression in various genetic backgrounds (liquid culture from BG11 to BG11$_0$)	
Wild type	9±0.9
WT/*PpetE-patX*	0
WT/*PpetE-patX-hetL*	13±0.95
WT/*PpetE-patXΔSS*	7.4±0.83
WT/*PpetE-patXΔSS/ PpetE*-alr1666*ΔSS*	0
patS and peptidase mutants (from BG11 plates to BG11$_0$ liquid)	
Wild type	9.42±0.32
ΔpatS	12±0.8
*ΔpatS Δ*all2656	12±1.7
*ΔpatS Δ*alr1666	37±2.1

Table S1: Percentage of heterocysts formed by different strains 48 hours after combined nitrogen starvation. The number of cells counted was 1000 on average. Observations were made on at least three independent cultures. Standard variations between different analyzed images are given.

Table S2: In silico prediction of signal-peptide-containing peptidases in *Nostoc*

MEROPS-ID	LocusID	Sec SP	Tat SP	CS Position	Predicted function
MER0081295	alr0975	0.998	0.000	CS pos: 24-25. Pr: 0.9779	Glutathione gamma-glutamylcysteinyltransferase
MER0016734	all5163	0.998	0.000	CS pos: 31-32. Pr: 0.9723	Cell wall cell division LysM type peptidase
MER0016737	all1862	0.998	0.000	CS pos: 35-36. Pr: 0.9669	Cell wall LytM homolog
MER0168590	all4822	0.999	0.000	CS pos: 22-23. Pr: 0.9781	Beta-lactamase
MER0016769	alr1666	0.999	0.000	CS pos: 27-28. Pr: 0.9755	Hypothetical
MER0016768	all2656	0.999	0.000	CS pos: 24-25. Pr: 0.9745	Hypothetical
MER0016178	alr3273	0.000	0.999	CS pos: 28-29. Pr: 0.8748	Peptidoglycan recycling, murein peptide carboxypeptidase
MER0039487	all5137	0.000	0.999	CS pos: 36-37. Pr: 0.7598	Caspase family protein

Table S4: *Nostoc* and *E. coli* strains

Strains	Description/genotype	Source/reference
***Nostoc* strains**		
Nostoc PCC 7120	Wild type strain (WT)	Pasteur Institute Collection
WT/P*petE-patX*	WT strain containing the pRL1272 P*petE-patX* plasmid	This study
WT/P*petE-patX* P*petE-hetL*	WT strain containing the pRL1272-P*petE-patX* plasmid and the pRL25T-P*petE-hetL* plasmid	This study
WT/P*petE-patXΔSS*	WT strain containing the pRL1272-P*petE-patX*Δ*SS* plasmid, where *patX* lacks the first 66 nucleotides, which encode the presumed Signal Peptide (SP)	This study
WT/P*petE*-alr1666*ΔSS*	WT strain containing the pRL25T-P*petE*-alr1666Δ*SS* plasmid, where alr1666 lacks the first 84 nucleotides, which encode the presumed Signal Peptide (SS)	This study
WT/P*petE-patXΔSS* P*petE*-alr1666*ΔSS*	WT strain containing the pRL1272-P*petE-patX*Δ*SS* plasmid and pRL25T-P*petE*-alr1666Δ*SS* plasmid	This study
WT/Palr1666-*gfp*	WT strain containing the pRR-gfp with alr1666 promoter	This study
WT/Pall2656*a-gfp*	WT strain containing the pRR-gfp with all2656 promoter a	This study
WT/Pall2656*b-gfp*	WT strain containing the pRR-gfp with all2656 promoter a	This study
*Δ*alr1666	*Nostoc* strain deleted from alr1666 gene	This study
*Δ*all2656	*Nostoc* strain deleted from All2656 and all6556 genes	This study
*Δ*alr1666*ΔpatS*	*Nostoc* strain deleted from alr1666 and *patS* genes.	This study
Δall2656 *ΔpatS*	*Nostoc* strain deleted from all2656 and *patS* genes.	This study
ΔpatX	*Nostoc* strain deleted from *patX* genes	This study
***E. coli* strains**		

BTH101	*F⁻, cya-99, araD139, galE15, galK16, rpsL1, hsdR2, mcrA1, mcrB1*	[5]
K-12 MG1655	F- lambda- *ilvG*- *rfb*-50 *rph*-1	Keo collection
MG1655 Δ*malE*	*malE* mutant of K-12 MG1655, Kanamycin resistant	Keo collection
BTH101/pKT25-*hetR*	BH101 containing the plasmid pKT25-*hetR*	[6]
BTH101/pUT18C-*hetR*	BTH101 containing the plasmid pUT18C-*hetR*	[6]
BTH101/pKT25-*patXΔSS*	BTH101 containing the plasmid pKT25-*patXΔSS*	This study
BTH101/pUT18C-*patXΔSS*	BTH101 containing the plasmid pUT18C-*patXΔSS*	This study
BTH101/pKNT25-*patXΔSS*	BTH101 containing the plasmid *patXΔSS*-pKNT25	This study
BTH101/pUT18-*patXΔSS*	BTH101 containing the plasmid *patXΔSS*-pUT18	This study
BTH101/pET28a-*his-hetR*	BTH101 containing the plasmid pET28a-*his-hetR*	[7]
Δ*malE*/p33tac-*malE*	DH5α containing the plasmid p33tac-*malE*	This study
Δ*malE*/p33tac-*malEΔSS*	DH5α containing the plasmid p33tac-*malEΔSS*	This study
Δ*malE*/p33tac-*patXSS1-malE*	DH5α containing the plasmid p33tac-*patXSS1-malE*	This study
Δ*malE*/p33tac-*patXSS2-malE*	DH5α containing the plasmid p33tac-*patXSS2-malE*	This study
Δ*malE*/p33tac- alr1666*SS* -*malE*	DH5α containing the plasmid p33tac-alr1666*SS*-*malE*	This study
Δ*malE*/p33tac- alr1666SS -*malE*	Δ*malE* containing the plasmid p33tac-alr1666*SS*-*malEΔSS*	This study
Δ*malE/* p33tac-*patX*	*malE* mutant containing the plasmid p33tacTrx-PatXΔss -MBP	This study
Δ*malE/* pBAD-alr1666	*malE* mutant containing the plasmid pBAD24-alr1666-V5	This study
Δ*malE/* pBAD-all2656	*malE* mutant containing the plasmid pBAD24-alr1666-V5	This study
Δ*malE/*p33tac-*patX* pBAD-alr1666	*malE* mutant containing the plasmids p33tac-*patX* and pBAD24-alr1666-V5	This study
Δ*malE/*p33tac-*patX* pBAD-all2656	*malE* mutant containing the plasmids p33tac-*patX* and pBAD24-all2656-V5	This study

Table S5: plasmids

Plasmids	Description	Source/reference
pKT25-zip	Two hybrid plasmid Kan^R	[1]
pUT18C-zip	Two hybrid plasmid Amp^R	[1]
pKT25	Two hybrid plasmid to have T25 at the N terminus Kan^R	[1]
pUT18C	Two hybrid plasmid to have T18 at the N terminus Amp^R	[1]
pKNT25	Two hybrid plasmid to have T25 at the C terminus Kan^R	[1]
pUT18	Two hybrid plasmid to have T18 at the C terminus Amp^R	[5]
p33tac	Expression plasmid in *E. coli* Cm^R	[8]
pRL1272	Replicative in *Nostoc* Ery^R	[9]
pSL2680	Replicating vector for constructing cpf1/CRISPR editing plasmids	Addgene(#85580)[10]
pKT25-*hetR*	pKT25 plasmid containing *hetR*	[6]
pRR duet GFP	Replicative plasmid in *Nostoc* bearing *gfp*	[11]
pUT18C-*hetR*	pUT18C plasmid containing *hetR*	[6]
pKNT25-*hetR*	pKNT25 plasmid containing *hetR*	This study
pUT18-*hetR*	Put18 plasmid containing *hetR*	This study
pKT25-*patXΔSS*	pKT25 plasmid containing *patXΔSS*	This study
pUT18C-*patXΔSS*	pUT18C plasmid containing *patXΔSS*	This study
pKNT25- *patXΔSS*	pKNT25 plasmid containing *patXΔSS*	This study
pUT18- *patXΔSS*-	pUT18 plasmid containing *patXΔSS*	This study
pBR1	pET28a-*his-hetR*	[12]

pRL25-*hetL*	pRL25T-P*petE-hetL*	[6]
pRL1272-*patX*	pRL1272-P*petE-patX*	This study
pRL1272-*patXΔSS*	pRL1272-P*petE-patXΔSS*	This study
pRL25-*patX*	pRL25T-P*petE-alr1666ΔSS*	This study
p33tac-*malE*	p33tac plasmid containing *malE*	This study
p33tac-*malEΔSS*	p33tac plasmid containing *malEΔSS*	This study
p33tac-*patXSS1-malEΔSS*	p33tac plasmid containing *patXSS1-malEΔSS*	This study
p33tac-*patXSS2-malEΔSS*	p33tac plasmid containing *patXSS2-malEΔSS*	This study
p33tac-*alr1666SS- malEΔSS*	p33tac plasmid containing *alr1666SS-malEΔSS*	This study
p33tac-*all2656SS- malEΔSS*	p33tac plasmid containing *alr1666SS-malEΔSS*	This study
pRRduet-alr1666 prom	pRRduet plasmid bearing a translational alr1666-*gfp* fusion	This study
pRRduet-all2656 prom a	pRRduet plasmid bearing a translational all2656-*gfp* fusion a	This study
pRRduet-all2656 prom b	pRRduet plasmid bearing a translational all2656-*gfp* fusion b	This study
pMB39	pAM5411 derivative vector for constructing cpf1/CRISPR editing plasmids	This study
p33tac-*patX*	p33tac plasmid bearing a *trx-patXΔSS-malE* fusion	This study
pBAD-alr1666-V5	pBAD24 plasmid containing alr1666ΔSS fused to the V5 tag	This study
pBAD-all12656-V5	pBAD24 plasmid containing all12656ΔSS fused to the V5 tag	This study

References

(1) Karimova, G.; Pidoux, J.; Ullmann, A.; Ladant, D. A Bacterial Two-Hybrid System Based on a Reconstituted Signal Transduction Pathway. *Proc. Natl. Acad. Sci. U.S.A.* **1998**, *95* (10), 5752–5756. https://doi.org/10.1073/pnas.95.10.5752.

(2) Zubay, G.; Morse, D. E.; Schrenk, W. J.; Miller, J. H. M. Detection and Isolation of the Repressor Protein for the Tryptophan Operon of *Escherichia Coli*. *Proc. Natl. Acad. Sci. U.S.A.* **1972**, *69* (5), 1100–1103. https://doi.org/10.1073/pnas.69.5.1100.

(3) Xu, X.; Risoul, V.; Byrne, D.; Champ, S.; Douzi, B.; Latifi, A. HetL, HetR and PatS Form a Reaction-Diffusion System to Control Pattern Formation in the Cyanobacterium Nostoc PCC 7120. *eLife* **2020**, *9*, e59190. https://doi.org/10.7554/eLife.59190.

(4) Rachedi, R.; Risoul, V.; Foglino, M.; Aoudache, Y.; Lang, K.; Champ, S.; Kaplan, E.; Orelle, C.; Douzi, B.; Jault, J.-M.; Latifi, A. Unravelling HetC as a Peptidase-Based ABC Exporter Driving Functional Cell Differentiation in the Cyanobacterium *Nostoc* PCC 7120. *Microbiol Spectr* **2024**, *12* (4), e04058-23. https://doi.org/10.1128/spectrum.04058-23.

(5) Karimova, G.; Pidoux, J.; Ullmann, A.; Ladant, D. A Bacterial Two-Hybrid System Based on a Reconstituted Signal Transduction Pathway. *Proc Natl Acad Sci U S A* **1998**, *95* (10), 5752–5756.

(6) Xu, X.; Risoul, V.; Byrne, D.; Champ, S.; Douzi, B.; Latifi, A. HetL, HetR and PatS Form a Reaction-Diffusion System to Control Pattern Formation in the Cyanobacterium Nostoc PCC 7120. *eLife* **2020**, *9*, e59190. https://doi.org/10.7554/eLife.59190.

(7) Roumezi, B.; Xu, X.; Risoul, V.; Fan, Y.; Lebrun, R.; Latifi, A. The Pkn22 Kinase of Nostoc PCC 7120 Is Required for Cell Differentiation via the Phosphorylation of HetR on a Residue Highly Conserved in Genomes of Heterocyst-Forming Cyanobacteria. *Front. Microbiol.* **2020**, *10*, 3140. https://doi.org/10.3389/fmicb.2019.03140.

(8) Bouillet, S.; Genest, O.; Méjean, V.; Iobbi-Nivol, C. Protection of the General Stress Response σS Factor by the CrsR Regulator Allows a Rapid and Efficient Adaptation of Shewanella Oneidensis. *Journal of Biological Chemistry* **2017**, *292* (36), 14921–14928. https://doi.org/10.1074/jbc.M117.781443.

(9) Elhai, J.; Wolk, C. P. A Versatile Class of Positive-Selection Vectors Based on the Nonviability of Palindrome-Containing Plasmids That Allows Cloning into Long Polylinkers. *Gene* **1988**, *68* (1), 119–138. https://doi.org/10.1016/0378-1119(88)90605-1.

(10) Ungerer, J.; Pakrasi, H. B. Cpf1 Is A Versatile Tool for CRISPR Genome Editing Across Diverse Species of Cyanobacteria. *Sci Rep* **2016**, *6* (1), 39681. https://doi.org/10.1038/srep39681.

(11) Rachedi, R.; Risoul, V.; Scholivet, A.; Foglino, M.; Latifi, A. Evidence That the PatB (CnfR) Factor Acts as a Direct Transcriptional Regulator to Control Heterocyst Development and Function in the Cyanobacterium *Nostoc* PCC 7120. *Molecular Microbiology* **2023**, *119* (4), 492–504. https://doi.org/10.1111/mmi.15044.

(12) Roumezi, B.; Avilan, L.; Risoul, V.; Brugna, M.; Rabouille, S.; Latifi, A. Overproduction of the Flv3B Flavodiiron, Enhances the Photobiological Hydrogen Production by the Nitrogen-Fixing Cyanobacterium Nostoc PCC 7120. *Microb Cell Fact* **2020**, *19* (1), 65. https://doi.org/10.1186/s12934-020-01320-5.
