# Supplementary figures and images for: Co-option of an ancestral peptidase controls developmental patterning in multicellular cyanobacteria
Source: iScience. 2025 Nov 28;29(1):114265. doi: 10.1016/j.isci.2025.114265 (PMC12756182; doi:10.1016/j.isci.2025.114265)

Figure 5B

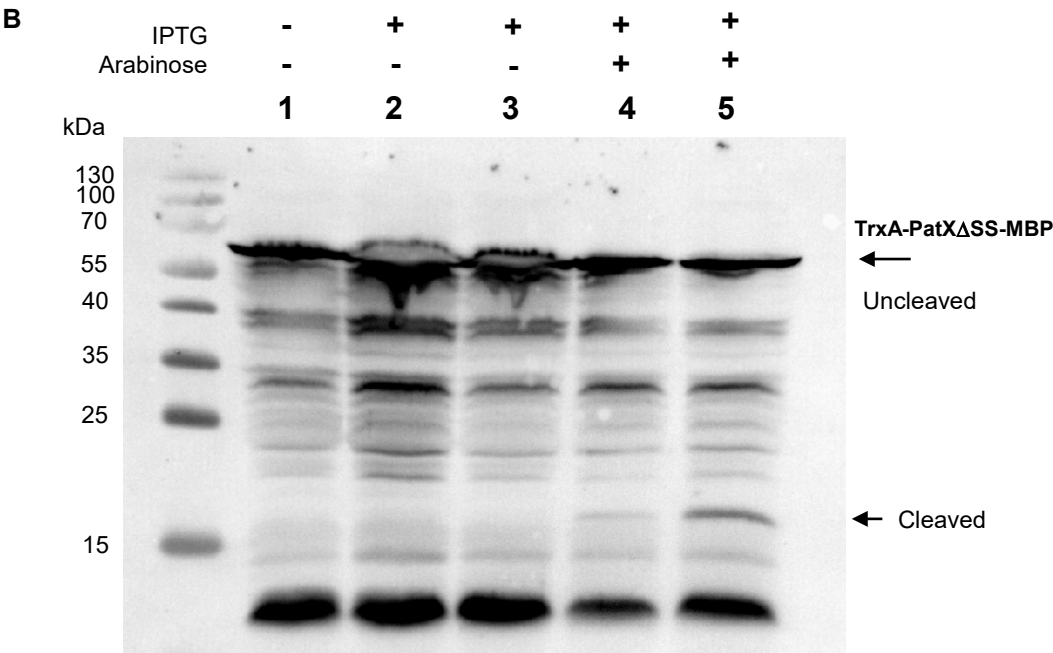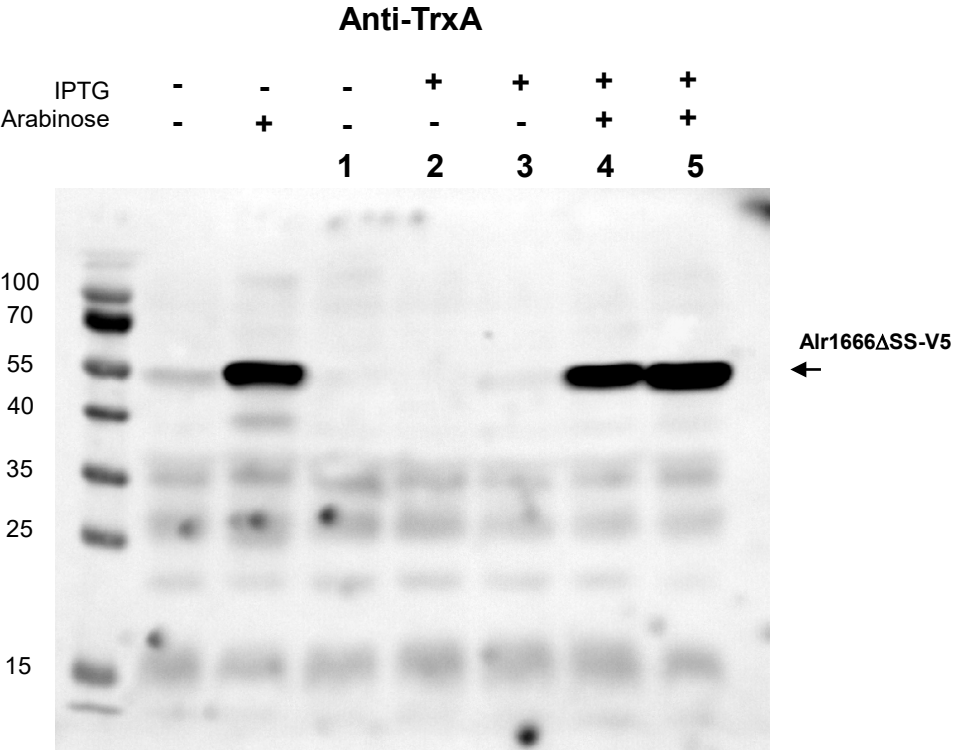

**Anti-V5**

Figure S5 A

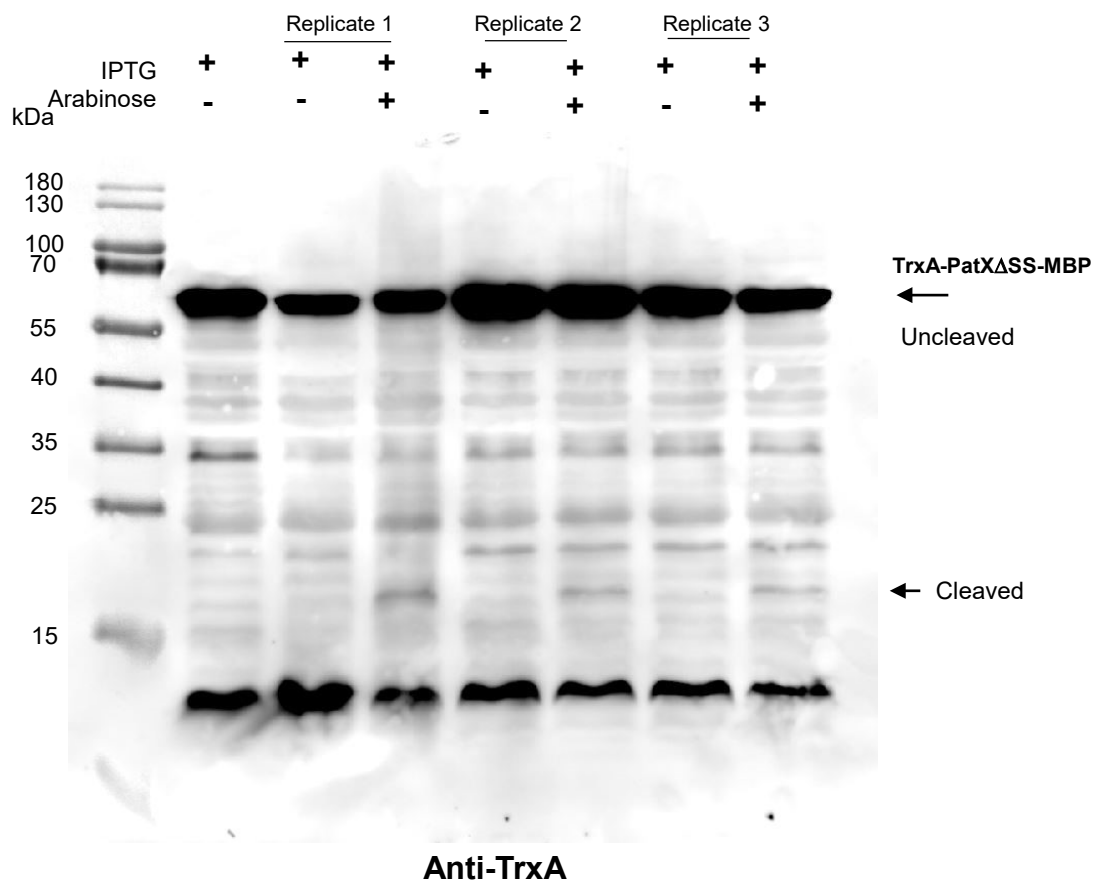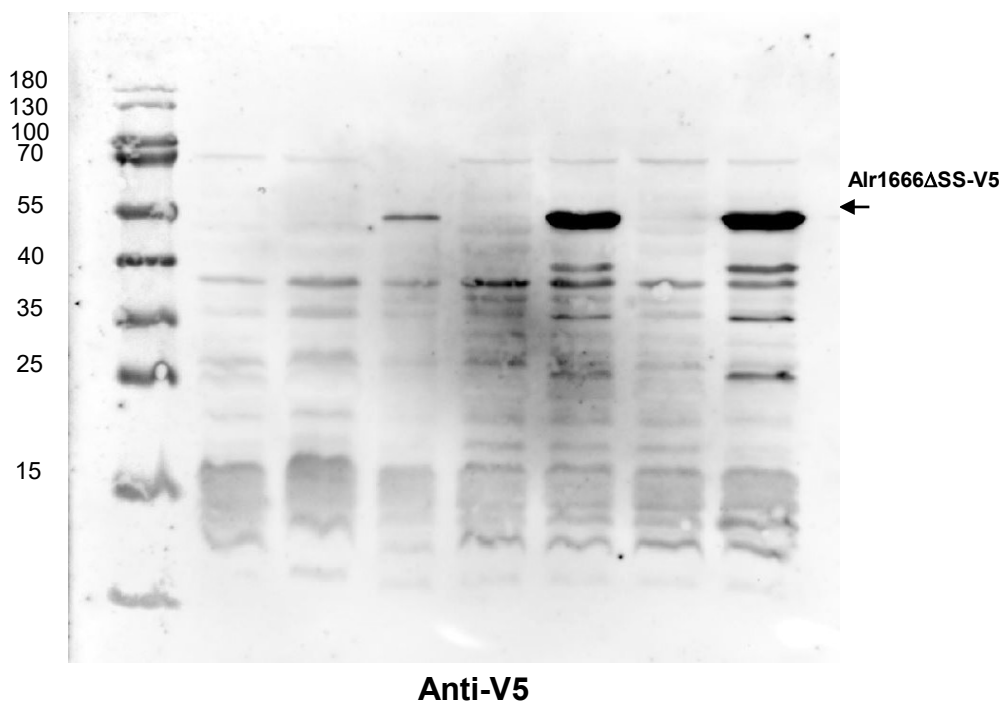

Figure S5 B

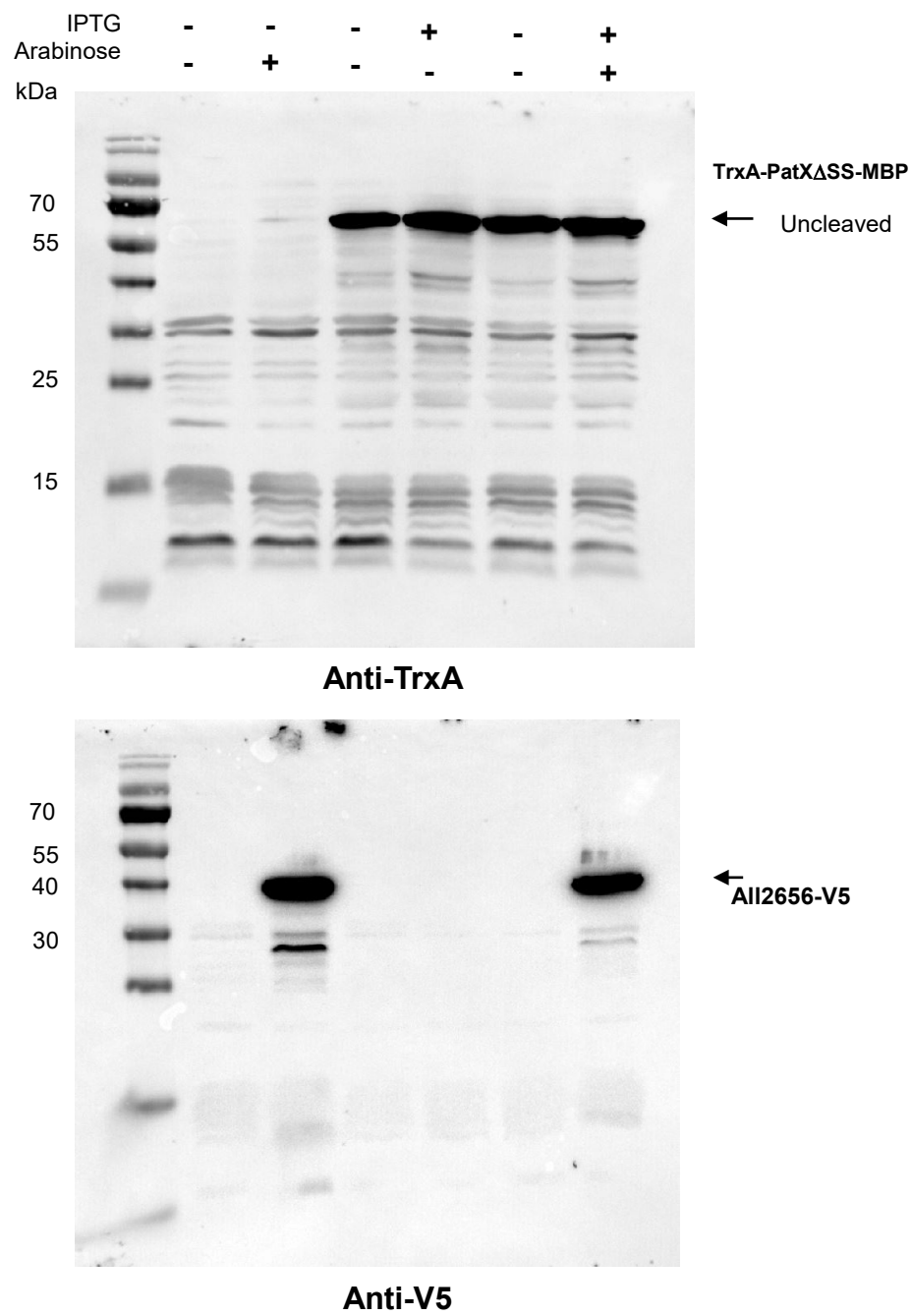

Figure S5 B

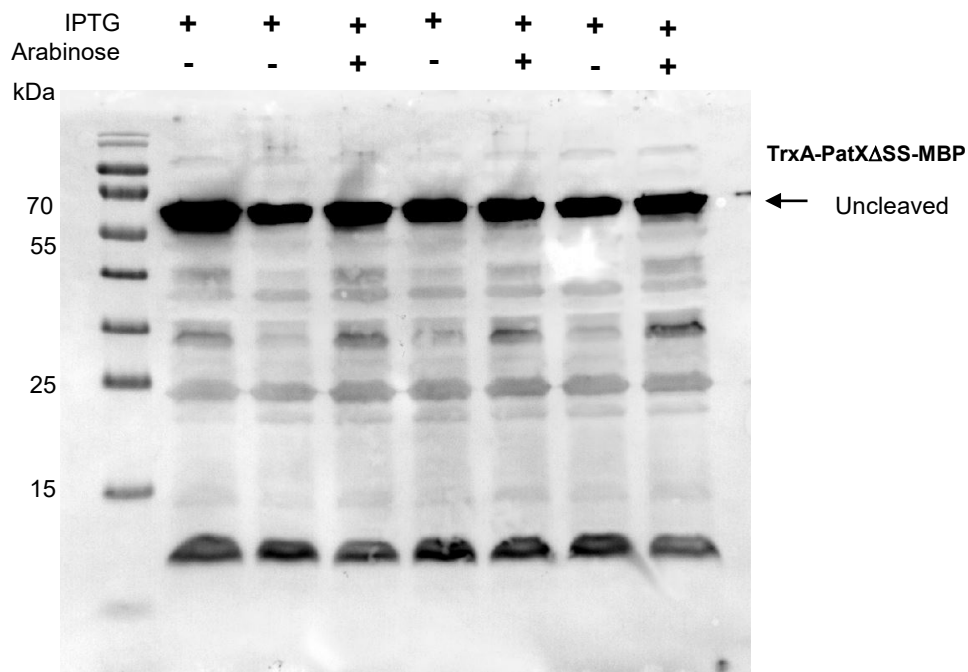

Anti-TrxA

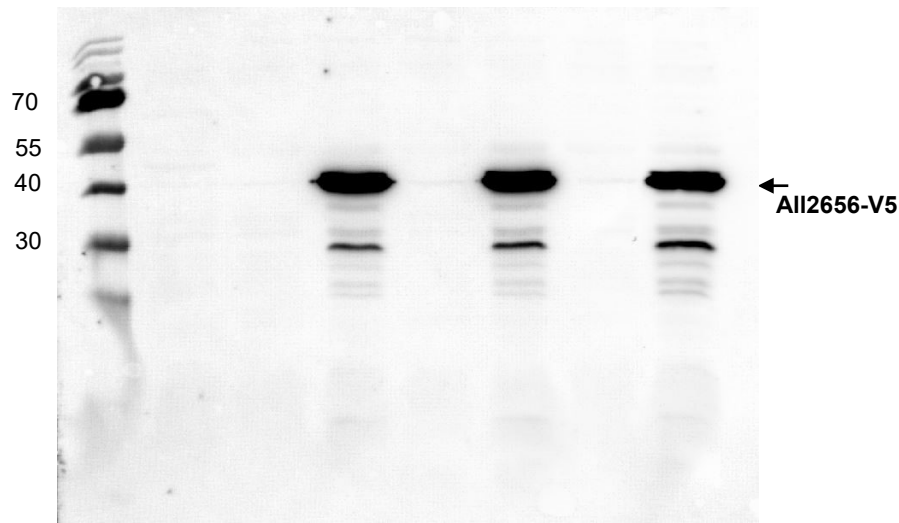

Anti-V5

Supplement: Data S1. Supplemental figures and tables [file mmc2.pdf]
